# Vanadium-Substituted Phosphomolybdic Acids for the Aerobic Cleavage of Lignin Models—Mechanistic Aspect and Extension to Lignin

**DOI:** 10.3390/ma13040812

**Published:** 2020-02-11

**Authors:** Louay Al-Hussaini, Franck Launay, Elena Galvez

**Affiliations:** 1 Centre National de la Recherche Scientifique, UMR 7197, Laboratoire de Réactivité de Surface (LRS), Sorbonne Université, F-75005 Paris, France; louayalhussaini@hotmail.fr; 2 Centre National de la Recherche Scientifique, UMR 7190, Institut Jean le Rond d’Alembert, Sorbonne Université, F-75005 Paris, France; elena.galvez_parruca@sorbonne-universite.fr

**Keywords:** keggin-type phosphovanadomolybdic acids, lignin, aerobic cleavage, parameter optimization, mechanistic study

## Abstract

This work deals with the aerobic oxidative cleavage of C-C and C-O bonds catalyzed by the Keggin-type phosphovanadomolybdic acid (H_6_[PMo_9_V_3_O_40_], noted H_6_PV_3_). The latter was synthesized by an adapted hydrothermal procedure classically used for lower vanadium content and was tested as a catalyst for the aerobic cleavage of 2-phenoxyacetophenone (noted **K1**_HH_) and 1-phenyl-2-phenoxyethanol (**A1**_HH_) used as two lignin models. The operative conditions (solvent, catalytic loading, etc.) were adjusted on **K1**_HH_ and extrapolated to **A1**_HH_. The cleavage of the alcohol model required more drastic conditions and therefore further optimization. Preliminary attempts on an Organosolv wheat straw lignin were performed too. From the kinetic study, high performance liquid chromatography (HPLC) and gas chromatography–mass spectrometry (GC-MS) data, a mechanism of the cleavage of both models was proposed.

## 1. Introduction

Lignin is the second most abundant natural polymer on our planet and the only natural, renewable and abundant source of aromatic compounds. It has raised increasing interest due to the depletion of oil, from which aromatic compounds are currently mainly produced. One of the most outstanding lignin valorization routes is its oxidation to aromatic platform molecules such as vanillin obtained from C-C cleavage [1]. Aerobic lignin cleavage in alkali media was already set up at the industrial scale on the 1930s. The major problem was the elimination of caustic wastes conjugated with a rather low yield of vanillin [2]. So, such of process is currently not competitive against the oil-based routes for vanillin production and has to be improved. In the present paper, O_2_ will be used as it is the most sustainable oxidant. However, it needs to be activated by a catalyst. Several metal-containing catalysts were assessed for the aerobic oxidative cleavage of lignin [3,4]. One of the most used metals is vanadium [5,6,7]. Keggin-type phosphovanadomolybdic acids (H_3+x_PMo_12−x_V_x_O_40_, nH_2_O) noted H_3+x_PV_x_ [8,9,10] are among the vanadium-based catalysts reported. For two decades, since the contributions of Weinstock et al. [11] and Evtuguin et al. [12], Keggin-type H_3+x_PV_x_ are well-known to be adequate for wood pulp oxygen delignification and more largely for aerobic oxidation [13]. Also, these catalysts already proved to be efficient for the C-C cleavage of α-ketols (room temperature) such as benzoin, a lignin model or cyclohexanone (70 °C) in an atmosphere of molecular oxygen [14,15,16].

**Figure 1:**
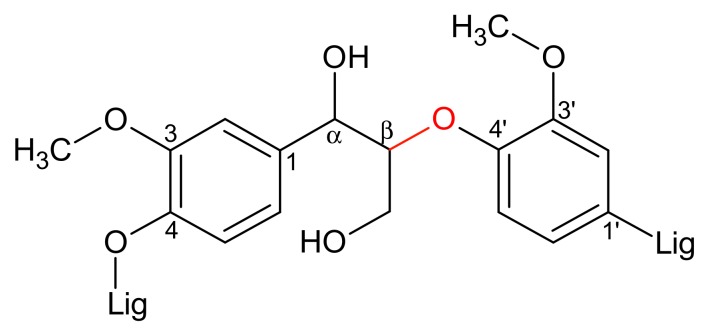
β-O-4′ bond in lignin polymer.

Since lignin is an extremely complex molecule, preliminary studies of its oxidative cleavage are usually carried out on models. Several linkages such as β-5, β-β and 5-5 are represented in lignin, but the β-O-4 bond (Figure 1) is always the major one in its native form [16]. As a result, many V-catalysts [17,18,19,20,21,22,23,24,25,26,27] were assessed for the aerobic oxidative cleavage of models bearing these linkages.

In the present contribution, the considered models are, on one hand 1-phenyl-2-phenoxyethanol noted **A1**_HH_ (already tested by Hanson et al. in presence of V^(+V)^(O)(dipic)(O^i^Pr), (dipic = dipicolinate)) and, on the other hand the ketone, 2-phenoxyacetophenone, **K1**_HH_, an oxidation product from the alcohol model. To the best of our knowledge, H_3+x_PV_x_ catalysts were never tested on such ketone models before. The active sites of H_3+x_PV_x_ are rather the vanadium addenda and it is known that the oxidative power of H_3+x_PV_x_ increases with vanadium content (mainly because of the reversible generation of pervanadyl cations VO_2_^+^ which are stronger oxidant than their parent H_3+x_PV_x_) [28,29]. However, an increase of vanadium degrades the stability of the H_3+x_PV_x_ [30] that may lead to loss of activity as observed by Odyakov et al. As a result, in this study, H_6_PV_3_ will be chosen as in the work of El Aakel et al. [14], because it seems to be the best compromise between oxidative power and stability. H_6_PV_3_ was synthesized through the hydrothermal pathway which just consists in refluxing the starting oxides and phosphoric acid in water without any addition of an external reagent [31] on the contrary to the most used etherate [32] (that involves a hazardous solvent [33] and strong acids) and oxo-peroxo [34,35] procedures usually used for such material. Already, such a pathway was attempted by Grate et al. for the synthesis of a H_6_PV_3_ 0.30 M in water. Even after seven days of reflux, the inclusion of vanadium was incomplete [36]. Therefore, in the present case, the procedure will be carried out in more diluted conditions (0.17 M) to favor the hydrothermal attack of V_2_O_5_.

## 2. Materials and Methods

### 2.1. Materials Synthesis

H_6_PV_3_ was synthesized through the attack of MoO_3_ (Acros Organics, Illkirch, France) and V_2_O_5_ (Sigma-Aldrich Chemie S.a.r.l., Saint-Quentin Fallavier, France) by H_3_PO_4_ (purity 85%, Carlo Erba, Val-de-Reuil, France) in refluxing water according to a procedure adapted from Kern et al. [31]. Hence, 0.39 g (3.3 mmol) of H_3_PO_4_ was dissolved in 200 mL of water and 3.9 g of MoO_3_ (27.3 mmol) and 0.92 g (5.1 mmol) of V_2_O_5_ were added.

### 2.2. H_6_PV_3_ Characterization

X-ray powder diffraction analyses (XRD, Advance D8, Bruker, Champs-sur-Marne, France) were performed using a Cu Kα radiation source without monochromator. The diffraction patterns were collected from 5° to 50°, with a scanning rate of 0.34° min^−1^. The crystalline phases were identified through the Rietveld analysis of the diffraction patterns (factors R_P_ and Chi_2_), using the Fullprof^®^ Suite software (program Fullprof. 2k, version 5.40, CEA, Saclay, France). Liquid ^31^P nuclear magnetic resonance analyses were performed on a 400 MHz apparatus (NMR, Bruker, Wissembourg, France). Hence, 30 mg of the solid was dissolved in 250 µL of D_2_O (Eurisotop) and 250 µL of H_2_O, then 7.5 µL of dioxane (SDS, Carlo-Erba, Val-de-Reuil, France) were added to the solution. For each analysis, 16 scans were recorded with a relaxation delay of 32 s. Inductive coupled plasma analyses (ICP) were carried out by Crealins (Lyon, Villeurbanne France) on an ICP Thermo-Fischer iCAM. ICP analysis allowed the determination of the value of x. Thermogravimetric analyses (TGA, Cp SDTQ600 system, TA Instruments, New Castle, USA) were performed in order to determine the hydration index of H_3+x_PV_x_, noted n. The temperature was increased to 600 °C at a heating rate of 10 °C/min under an air flow of 100 mL min^−1^.

### 2.3. Catalytic Tests

The efficiency of H_6_PV_3_ for the aerobic cleavage in atmospheric conditions was tested on **K1**_HH_ (0.32 g, 1.5 mmol), **A1**_HH_ (0.32 g, 1.5 mmol) or the acetate derivative of **A1**_HH_, **Est^α^A1**_HH_ (0.41 g, 1.5 mmol). The preparation procedure of all these compounds is detailed in the Supporting Information. The substrate, and then H_6_PV_3_ (0–88 mg), were dissolved in 15 mL of the solvent in a Schlenk tube connected to a gas burette system to monitor the molecular oxygen consumption. The Schlenk tube was purged with molecular oxygen and then heated during 24 h.

Also, some tests on **A1**_HH_ were carried out at higher pressure. In a typical experiment, 0.54 g of the substrate (2.5 mmol) and 61.4 mg of H_6_PV_3_ (Mo + V 15 mol%) were dissolved in 25 mL of solvent (MeCN + 10 vol% AcOH) in a Teflon container that was further placed in a 100 mL autoclave. The mixture became instantaneously green due to the reduction of the catalyst. Once the autoclave was sealed, the reaction medium was purged three times by molecular oxygen at room temperature. The pressure was set to 5 bar and then heated at 80 °C or 120 °C under stirring. Once the reaction was completed, the recovered solution is orange-brown.

### 2.4. Monitoring of the Tests

After cooling the reaction mixture, 0.5 mL was sampled and dropped in a 20 mL volumetric flask and diluted by H_2_O-MeOH-AcOH 49:49:2 for high performance liquid chromatography (HPLC) analysis (LC-20AD, Shimadzu France, Marne-la-Vallée, France) using a Shim-pack column (4.6 × 100 mm, 2.2 µm) heated to 40 °C in a CT0-10AS oven. The mobile phase was constituted of aqueous AcOH (0.5 vol%) and methanol (HPLC grade, VWR Chemicals) used at a flow rate of 0.4 mL min^−1^ (0–13 min: methanol 40%, 13–17 min: gradient from 40 to 60% of methanol, 17–33 min: methanol 60%, 33–37 min: gradient from 60 to 40% of methanol and 37–50 min: methanol 40%). The compounds were detected by an SPD-M20A UV detector (210, 220 and 254 nm (Figure S1)). Benzaldehyde, benzoic acid and phenols were quantified according calibration curves (see Supporting Information, Figure S2). A typical example is given on the Supporting Information (Figure S1). Benzaldehyde, benzoic acid and phenol were quantified according calibration curves (see Supporting Information, Figure S2 and Table S1). The other products (of the C-O cleavage such as phenylglyoxylic acid as well as quinones and so forth, detected by HPLC (see Figure S1)) were not taken into account.

The reaction medium was also analyzed without dilution by gas chromatography–mass spectrometry (GC-MS) in duplicate on a GC-2010 Plus gas chromatograph using a HP-5MS ((5%-phenyl)-methylpolysiloxane) column. The temperature of the injector was set to 250 °C. The oven temperature raised from 70 °C to 250 °C with a ramp of 5 °C min^−1^ and the temperature was held at 250°C during 10 min. Helium (1.5 mL min^−1^) was used as the carrier gas. The products were detected by a GCMS-QP2010 SE mass spectrometer with a time delay of 4 min (Table S2). The ion source and the mass detector were heated to 200 and 250°C respectively and the voltage was 0.2 kV. The mass spectra were recorded for 35 < m/z < 300 every 0.3 s.

### 2.5. Preliminary Tests on an Organosolv Lignin

These tests were carried out on a purified wheat straw Organosolv lignin (extracted from wheat straw according to a patent FR 2926824 [37] and purified according to Mbotchak et al. [38]) in the presence of H_6_PV_3_ in the conditions of **A1**_HH_ cleavage. The targeted products were *p*-hydroxybenzaldehyde, vanillin, syringaldehyde and their corresponding acids. Experiments were carried out directly with 5 bars of molecular oxygen at 120 °C. Hence, 0.85 g of lignin and 59.6 mg of H_6_PV_3_ (Mo + V 15 mol% according to the substrate) were weighted and contacted with 25 mL of MeCN − 10 vol% AcOH in an autoclave. The procedure used was then similar to that of **A1**_HH_ oxidation under pressure. At the end of the reaction, since lignin is barely soluble, the mixture had to be filtered on a sintered filter (porosity n 4). An aliquot of the filtrate (20 µL) containing the targeted phenolic aldehydes and acids was poured in a test tube capped by a septum in order to evaporate the solvent. Then, 20 µL of pyridine, 10 µL of a solution of an internal standard (biphenyl, 10 mM in acetonitrile) and 250 µL of BSTFA (N,O-Bis(trimethylsilyl)trifluoroacetamide) were added and the mixture was stirred overnight. Afterwards, the obtained solution was analyzed by GC-MS in duplicate.

## 3. Results

H_6_PV_3_ was characterized by XRD and liquid ^31^P NMR to validate the Keggin-type structure and to obtain its yield. Then, the number of vanadium equivalents and the hydration index noted x and n were determined by ICP and thermogravimetric analysis (TGA) respectively. H_6_PV_3_ was then tested for **K1**_HH_ cleavage. Meanwhile, the operative conditions were optimized. Then, H_6_PV_3_ was assessed in optimized conditions for **A1**_HH_ and then an Organosolv wheat straw lignin.

### 3.1. H_6_PV_3_ Synthetic Procedure

**Figure 2:**
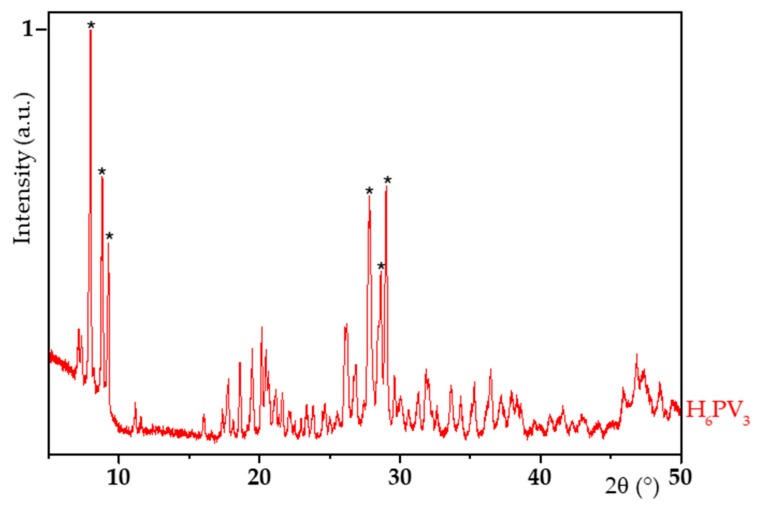
X-ray diffraction (XRD) profile of H_6_PV_3_ (main characteristic peaks marked by * symbol).

H_6_PV_3_ synthesized using the hydrothermal pathway was characterized by XRD and ^31^P NMR. The X-Ray profile of H_6_PV_3,_ as well as of its starting oxides acquired in the same conditions, are given on Figure 2.

The diffractogram of H_6_PV_3_ was shown to be very similar to that of tridecahydrated phosphomolybdic acid, H_3_PMo_12_O_40_, 13 H_2_O (JCPDS 00-043-0317, see Figure S3) which adopts a triclinic ($P\bar{1}$) structure. The main characteristic peaks of this Keggin-type structure are expected at *c.a.* 9° and *c.a.* 28°. It has to be noted that the peaks at *c.a.* 9° are not present in the profiles of the starting oxides and are clearly related to the triclinic structure of V_3_. However, H_6_PV_3_ turned out to be constituted of at least two crystalline phases since R_P_ = 15.1% and Chi_2_ = 13.1% (Table 1). The second crystalline phase could be unreacted vanadium oxide or a H_6_PV_3_ having a different hydration index. The cell parameters of the main phase of H_6_PV_3_ were calculated on Fullprof^®^ and are given in Table 1 (an extract of Fullprof is given on Figure S4).

**Figure 3:**
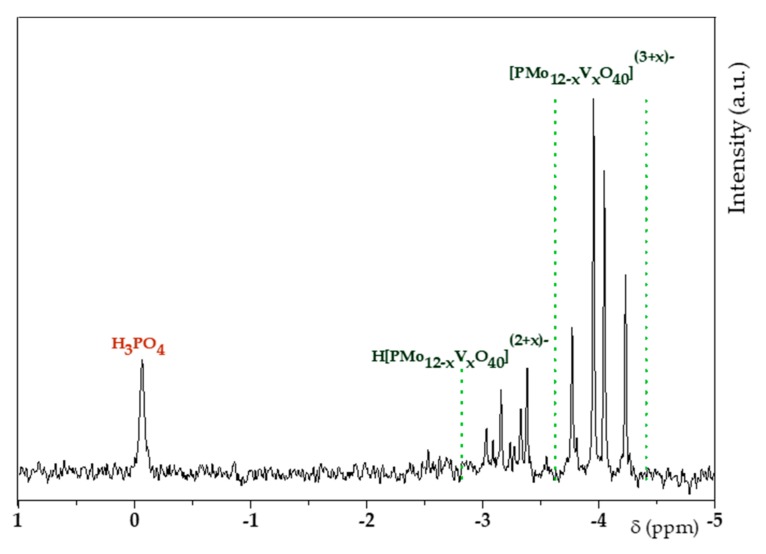
^31^P nuclear magnetic resonance (NMR) profiles of V_3_.

Liquid ^31^P NMR (Figure 3) was performed in order to establish the composition of H_6_PV_3_ in solution. To do this, the structure of H_6_PV_3_ had to be stabilized by dioxane [39] at a final pH of about 1.

The spectrum of H_6_PV_3_ was more complex compared to those of H_3+x_PV_x_ having lower amounts of V addenda [36,40]. On one hand, 13 isomers (taking account only the α isomers) can be envisaged and, on the other hand, the introduction of vanadium leads to a weaker acidity strength [40]. As a result, the additional peaks from −2.9 to −3.25 ppm observed for H_6_PV_3_ could be associated to anionic and protonated forms of H_6_PV_3_ (pK_a_ ≈ 3). Also, pervanadyl cations (VO_2_^+^) are known to be more easily ejected from the Keggin-type sphere leading to lacunar H_3+x_PV_x_ among others [28]. NMR enabled to confirm the Keggin-type structure. As shown on Figures S5 and S6, the maximal relaxation time was established to 1.02 ± 0.11 s. So, the relaxation time set in each acquisition was higher than 5 times the relaxation delay of every phosphorous nucleus. As a result, the integration was considered to be quantitative and, because reactants were added in stoichiometric amounts, the area of the peak at 0 ppm enabled to evaluate the amount of unreacted H_3_PO_4_ and so the H_3+x_PV_x_ yield, assuming that H_3_PO_4_ was the only by-product. The value, hence calculated, was 79%.

**Figure 4:**
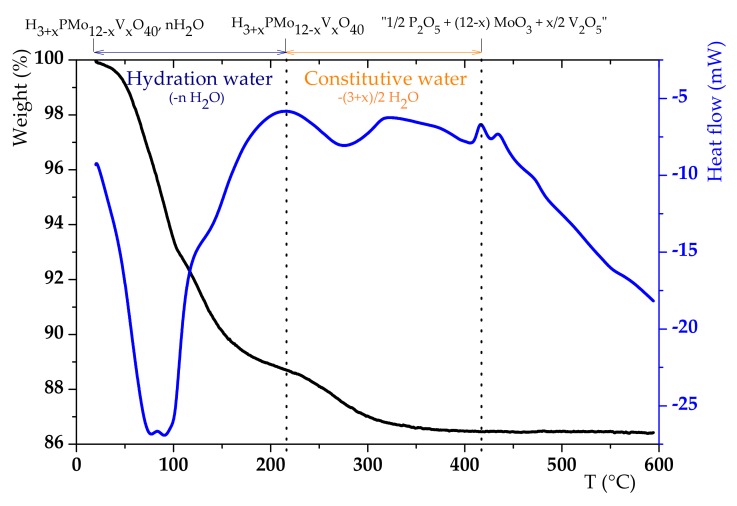
Thermogravimetric analysis (TGA)–differential scanning calorimetry (DSC) profile of H_6_PV_3_.

Once the Keggin-type structure validated by XRD and confirmed by liquid ^31^P NMR, the number of vanadium equivalents in H_6_PV_3_ solid was then calculated from the weight fraction of molybdenum (46.16 wt%) and vanadium (7.06 wt%) obtained by ICP. It was shown to be equal to 2.680 ± 0.005 (vs a theoretical × of 3). The hydration index n calculated by thermogravimetric analysis and differential scanning calorimetry (TGA-DSC was 13 (Figure 4) through the first weight loss [41], which is in good agreement with the XRD data. So, the general formula for H_6_PV_3_ is H_5.68_PMo_9.32_V_2.68_O_40_, 13H_2_O. The second weight loss weight loss may enable to calculate the number of vanadium equivalents, but, here, the loss of hydration and constitutive water are overlapped [42].

### 3.2. **K****1**_HH_ aerobic cleavage

**Figure 5:**
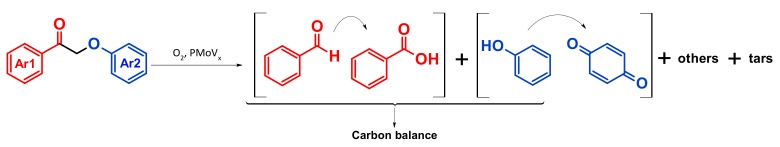
Cleavage of **K1**_HH_ by O_2_.

Firstly, H_6_PV_3_ was assessed for the aerobic cleavage of **K1**_HH_ under atmospheric pressure of oxygen in pure solvents. The solution was heated to the targeted temperature during 24 h. In such conditions, phenol (PhOH), benzaldehyde (PhCHO) and benzoic acid (PhCOOH) were obtained as the main products (Figure 5).

The amount of *p*-quinone could not be determined accurately but the latter was rather small. The conversion of **K1**_HH_, the yields of the main products as well as the carbon balance values (CB) are given in Table 2. The products from both C-C (PhCHO and PhCOOH) and C-O (PhOH) cleavage and the starting material were taken into account in the calculation of the carbon balance. As usually observed, the carbon balance tends to decrease with increasing conversion as the result of the occurrence of side reactions. It has to be noted here that, the maximum value of the carbon mass is normally lower than 100%. Indeed, C_1_ products such as formaldehyde, formic acid, CO_2_ and so forth, coming from the transformation of the CH_2_ group of **K1**_HH_ could not be detected quantitatively and were not taken into account. Assuming all of this, CB should not take values higher than $CB_{max}\left(\%\right)=\frac{1400-Conv\;\left(\%\right)}{14}$.

Several solvents were tested. Refluxing conditions (excepted for the test in pure acetic acid) in presence of H_6_PV_3_ (Mo + V 15 mol%) during 24 h were applied. As a starting point, methanol (Table 2, entry 1) and acetic acid (entry 3) were used like El Aakel et al. did [18], for the C-C bond cleavage of cyclic α-hydroxyketones in presence of V_3_. Acetonitrile was assessed too (Table 2, entry 2). The conversion of **K1**_HH_ in refluxing acetonitrile (Table 2, entry 2) was not so high (48%, brown) and turned out to be lower in refluxing methanol (14%, green, entry 1). The highest conversion of **K1**_HH_ and yields of PhOH and PhCOOH were obtained in acetic acid (Table 2, entry 3). However, lignin is rich of alcohol moieties that may be esterified by acetic acid, giving rise to a lignin more recalcitrant to oxidation [43]. Consequently, in spite of these results, acetonitrile was preferred to acetic acid provided that some further optimization would be done.

Mixtures of acetonitrile and acetic acid with varying AcOH concentrations (0 ≤ z ≤ 20% AcOH in volume) have also been tested as solvents at 82 °C (Table 2, entries 4–8) [44,45,46]. The conversion of **K1**_HH_ as well as the yields of phenol coming from C-O and of benzoic acid from C-C cleavage, respectively raised with the acetic acid content. A z value of 10 vol. % was found to be an optimum. Indeed, at 20 vol. % (Table 2, entry 8 vs 6), the carbon balance decreased (72% vs 80%). Since C-O bond is weaker than C-C bond, its cleavage was favored giving rise to an enhanced phenol production compared to benzaldehyde and benzoic acid. Some minor products could be detected by HPLC like phenylglyoxylic acid for z = 2.5 (yield 2.7%, estimated by HPLC) and z = 5 (yield 4.5%). Furthermore, benzaldehyde yield was found to be always lower than those of phenol and benzoic acid. The maximum yield of benzaldehyde was reached for z = 5 (Table 2, entry 5) and the PhCHO/PhCOOH ratio was maximized for z = 2.5 (Table 2, entry 4) and tends to decrease for a higher content of acetic acid until the disappearance of benzaldehyde in pure acetic acid (Table 2, entry 3). So, acetic acid appears to favor the benzaldehyde over-oxidation.

The test in MeCN − 10 vol% AcOH was repeated at 65 °C (Table 2, entry 7). From the comparison with the test in methanol (Table 2, entry 1), the test in acidified acetonitrile gave rise to better conversion and yields of cleavage products. The (PhCHO + PhCOOH)/PhOH ratio was equal to 1.2 at 65 °C and 1 at 80 °C.

A series of experiments were performed in order to evaluate the influence of the H_6_PV_3_ loading (from 7 to 36 mol% of Mo + V, Table 2, entries 6, 9–11). Logically, the higher the metal loading, the higher the conversion of the substrate. At low Mo + V loading (Table 2, entry 10, 7 mol%), the conversion was rather low (43%). At the highest metal loading (Table 2, entry 11, 36 mol%), the conversion was expectedly higher but the carbon balance tended to be rather low (58% only, compared to 80% for 15 mol% Mo + V). For these reasons, a metal loading of 15 mol% was found to be a good compromise. Besides, in the case of the tests performed at high metal loading (Table 2, entry 11), the oxygen consumption per mole of converted substrate was slightly lower probably because the catalyst might behave as an oxygen donor [47]. Materials such as H_6_PV_3_ are characterized by rather strong acidic properties, that is the reason why we have decided to check also the influence of the acidity on the cleavage of **K1**_HH_. This was done owing to a reference test (no metal) performed with H_2_SO_4_ (Table 2, entry 9) which showed that the conversion of **K1**_HH_ and especially the yields of PhCHO and PhCOOH were lower than with V_3_. Phenol was by far the main product meaning that the C-C cleavage requires the presence of a metallic catalyst whereas C-O linkage may be cleaved more easily, just by acidity.

**Figure 6:**
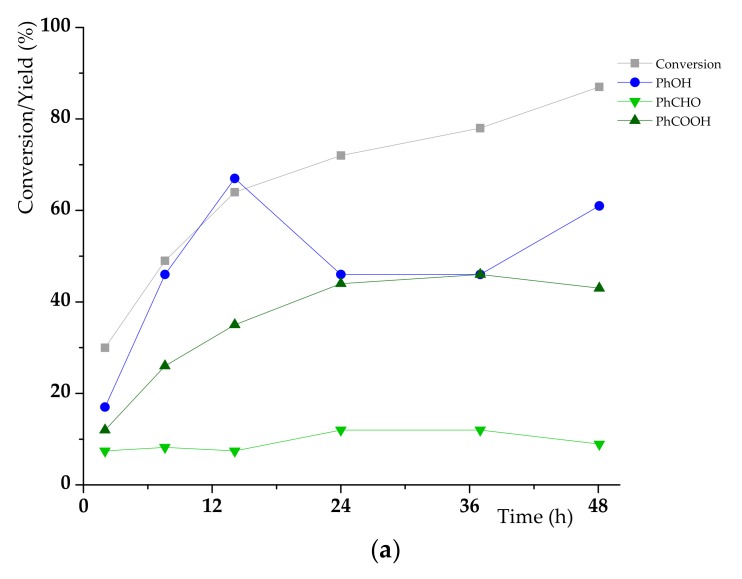

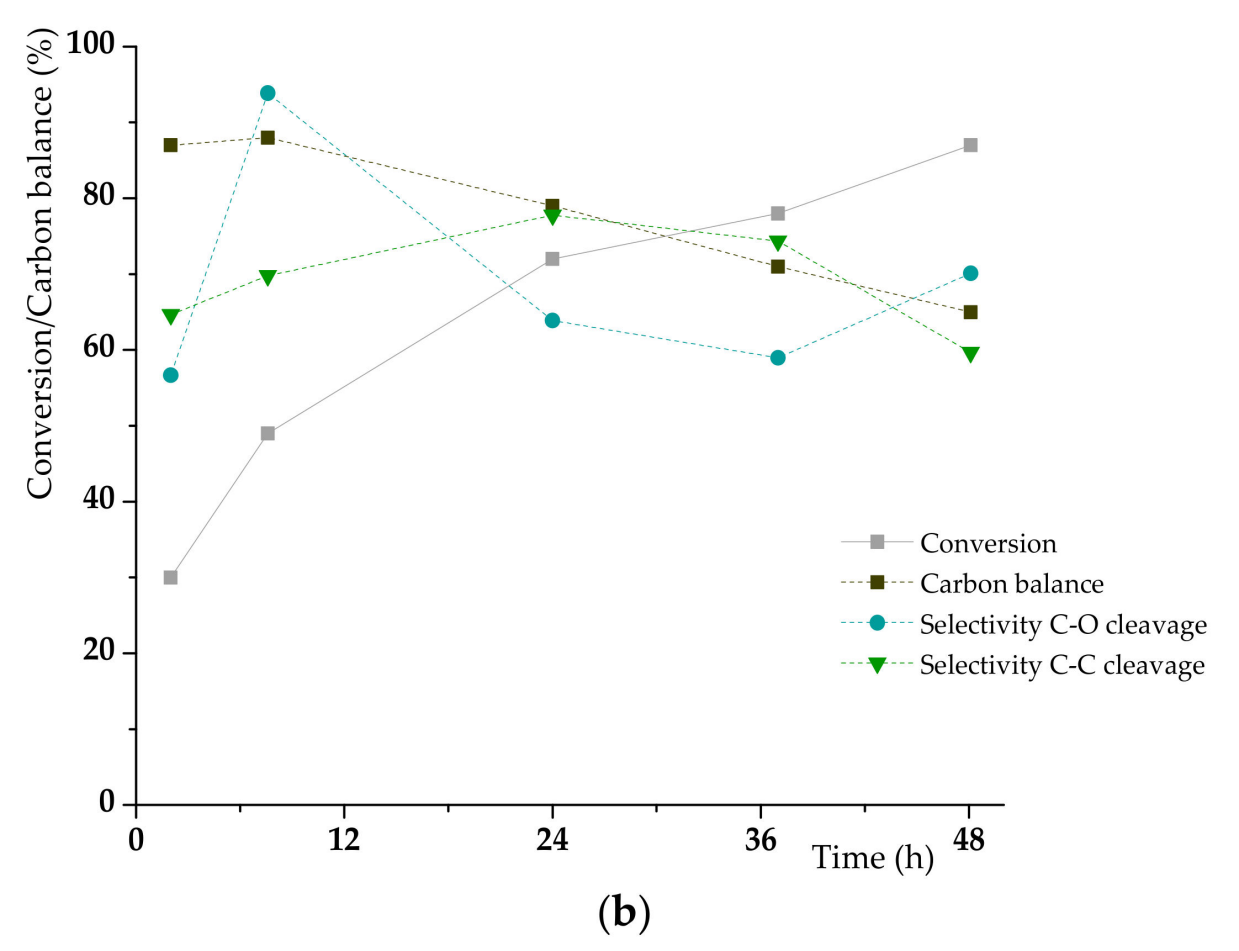
Evolution of different reaction parameters vs time for the H_6_PV_3_-catalyzed aerobic cleavage of K1HH; (**a**) yield of cleavage products, (**b**) carbon balance and selectivity for C-O and C-C cleavage products.

Most of the previous tests in MeCN − 10 vol% AcOH were performed at 80 °C within 24 h. Here, we propose to monitor, during 48 h, the **K1**_HH_ conversion, the carbon balance as well as the yields of the three main products in our reference conditions, that is, **K1**_HH_ 100 mM, H_6_PV_3_ (Mo + V 15 mol%), MeCN − 10 vol% AcOH (15 mL), T = 80 °C (see Figure 6). The yield of *p*-quinone was also calculated, though with a low precision. As expected, the conversion of **K1**_HH_ raised whereas the carbon balance decreased throughout the reaction. The yield of PhCHO increased until 24 h with a maximum of 12%, then it slowly decreased to 9%. PhCOOH production reached also its maximum (46%) within 37 h. After, it did not change significantly. First, before 24 h, the reaction duration was positive for PhOH formation with a maximum of 67%. Then, due to over-oxidation into *p*-quinone as observed after 24 h, the yield of PhOH decreased until 46% till 37 h. This led to a significant decrease of the carbon balance. For short reaction times (<24 h), the (PhCHO + PhCOOH)/PhOH ratio was often lower than 1 meaning that PhOH is produced in greater extent than the two other main products. As a result, it is highly probable that part of phenol was issued from C-O cleavage only. The detection by HPLC of C_8_-products such as phenylglyoxylic acid confirms such hypothesis. The yield of the latter could be estimated to *c.a.* 6% showing no significant evolution between 37 and 48 h. Hence, the quinone production was limited and took place to a much smaller extent compared to phenol formation from **K1**_HH_. Furthermore, no molecular oxygen volume variation was observed between 37 and 48 h while the conversion of **K1**_HH_ still raised in the meantime. Otherwise, the molecular oxygen consumption per mole of converted substrate was quite constant with a value of 1.5 mol of O_2_ per mole of consumed **K1**_HH_.

Besides, the quantity of gaseous molecular oxygen is much higher than dissolved molecular oxygen. So, it can be hypothesized that dissolved oxygen is constantly renewed and so, the concentration of molecular oxygen is constant (order degeneration). We tested a general expression $\left(-\frac{d\left[K1_{\mathrm{HH}}\right]}{\mathrm{dt}}=k_{\mathrm{app},K}{\left[K1_{\mathrm{HH}}\right]}^{q_{K}}\right)$ with q_K_ order values different from 1 and it appeared that the best modeling of the experimental data corresponds to [**K1**_HH_]^−0.25^ = f(t) (Figure S7) curve, that is, (after derivation) q_K_ = 1.25, which is close to 1.

### 3.3. **A1**_HH_ aerobic Cleavage

**Figure 7:**
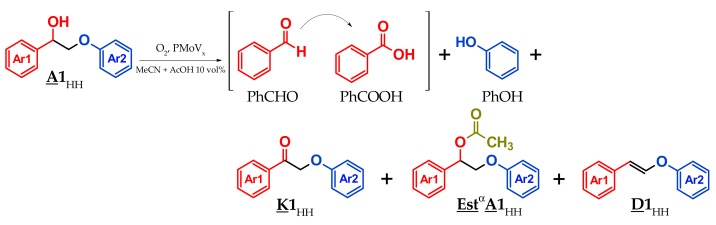
Aerobic oxidation of **A1**_HH_ by O_2_.

**K1**_HH_ is not the best model of lignin. **A1**_HH_ has a structure closer to lignin and will be now considered as the substrate (Figure 7).

**A1**_HH_ was tested first in the conditions optimized for **K1**_HH_, that is, in the presence of H_6_PV_3_ (Mo + V 15 mol%) in MeCN + 10 vol% AcOH under atmospheric pressure of O_2_ (Table 3, entries 1–2).

Undoubtedly, **A1**_HH_ proved to be more resistant than **K1**_HH_. Under atmospheric pressure, the oxidation of the alcohol function was favored as well as the formation of the esterification (by acetic acid) and dehydration products, noted **Est**^α^**A1**_HH_ and **D1**_HH_, respectively. In the presence of H_2_SO_4_, instead of H_6_PV_3_ (Table 3, entry 2), the conversion fell to 9.1% (vs 35% in presence of V_3_, Table 3, entry 1), the yield of phenol was 1.9% (vs 4.6%) and benzoic acid was not detected. Also, the ester was tested in the same conditions in the presence of H_6_PV_3_. A conversion of 7% and only traces of cleavage products were obtained meaning that the esterification is a dead-end reaction. Therefore, such a reaction has to be mitigated.

**Figure 8:**
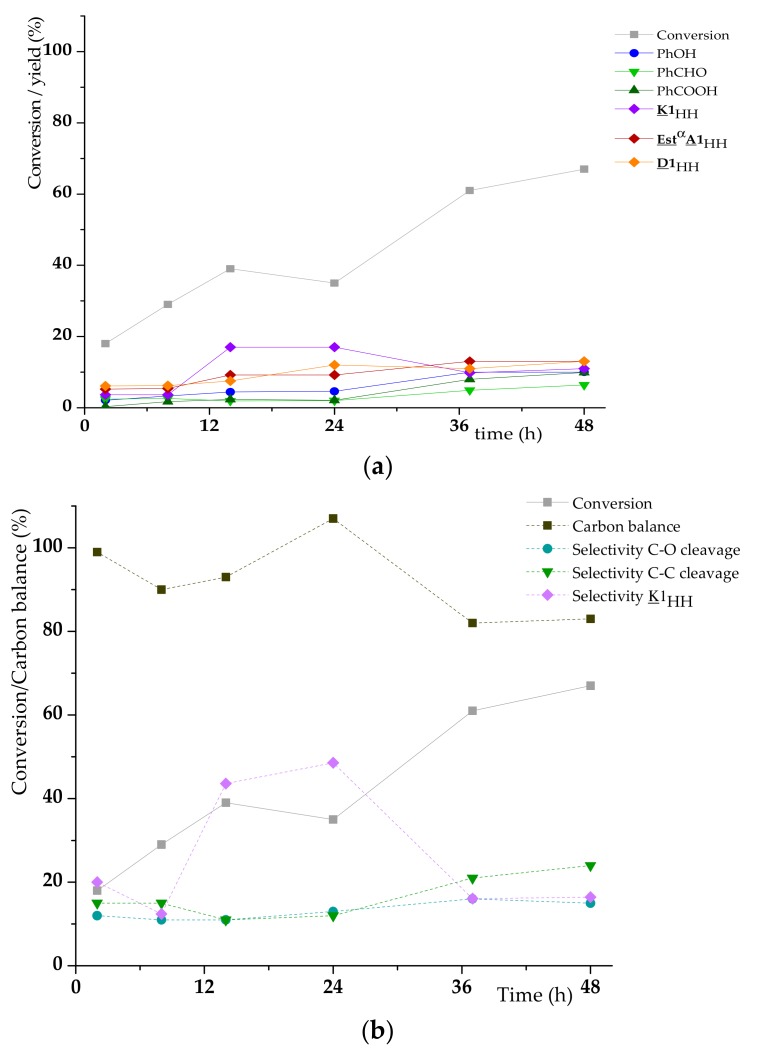
Evolution of different reaction parameters vs time for the H_6_PV_3_-catalyzed aerobic cleavage of **A1**_HH_; (**a**) yield of cleavage products, (**b**) carbon balance and selectivity for C-O and C-C cleavage products (see conditions in Table 3).

The cleavage reaction of **A1**_HH_ was also monitored for 48 h (Figure 8). During that time period, the conversion of **A1**_HH_ raised until 67% (instead of 35% within 24 h). Clearly, the oxidation of **A1**_HH_ was slow compared to that of **K1**_HH_ under the same conditions. The yields of phenol and benzoic acid raised both until 10% and benzaldehyde yield was stable from 8 to 24 h meaning that its production and its oxidation took place at very similar rates. **K1**_HH_ seemed to accumulate (the yield of **K1**_HH_ raised until 17% at 24 h, then the latter was converted since its yield was 11% after 48 h showing that, in presence of **A1**_HH_, H_6_PV_3_ becomes less efficient for the aerobic oxidative cleavage of **K1**_HH_. The side products **Est**^α^**A1**_HH_ (calibrated at 254 nm, see Figure S2) and **D1**_HH_ (identified by ^1^H NMR) accumulated during the course of the reaction. With increasing time, the phenol selectivity was stable whereas the selectivity in C-C cleavage increased. The carbon balance decreased after 37 h meaning that side reactions were also accelerated.

Similarly to **K1**_HH_ cleavage, the rate law for **A1**_HH_ can be written $-\frac{d\left[\underset{–}{A}1_{\mathrm{HH}}\right]}{\mathrm{dt}}={\;k}_{\mathrm{app},A}{\left[\underset{–}{A}1_{\mathrm{HH}}\right]}^{\left(q_{A}\right)}$. Since the best fit with the experimental data (max. R² = 0.9999) was obtained with [**A1**_HH_]^−0.7^ = f(t) (Figure S7), this gave, after derivation, q_A_ = 1.7. The values of k_app,A_ (0.0009 M^−0.25^ s^−1^) and k_app,K_ (0.0036 M^−0.7^ s^−1^) were also determined (Figure 8a–b and Figure S7). From these results, a slower kinetics of **A1**_HH_ (vs **K1**_HH_) conversion and a higher impact of the substrate concentration in the case of **A1**_HH_ could be evidenced.

More stringent conditions had to be implemented in order to favor the formation of oxidative cleavage products. In a first attempt, the O_2_ pressure was raised from 1 to 5 bar without modifying the temperature (80 °C; Table 3, entry 3). Such rise of O_2_ pressure favored the conversion (54 vs 35%, Table 3, entry 3 vs 1) as well as the yield of PhCHO and PhCOOH, that is, the selectivity for C-C cleavage products. Nonetheless, dehydration and esterification took place at a higher extent. Since then, the duration of the test had to be shortened to 6 h (entry 4 vs 3) instead of 24 h and **A1**_HH_ conversion decreased, as expected, from 54 to 33%.

Another set of conditions was tested. Hence, the temperature was raised to 120 °C and the duration was shortened to 2 h (Table 3, entry 5) to preserve the carbon balance. After 2 h, the conversion was 44% (vs 33% at 80°C after 6 h, Table 3, entry 4). The temperature increase turned out to have a positive impact (Table 3, entry 5 vs 4) on the selectivity for the C-O (16% vs 11%) and C-C cleavage (32% vs 19%). Moreover, **Est**^α^**A1**_HH_ (11% vs 9.9%) was produced at similar extent and the carbon balance was not significantly degraded (96% vs 99%). Therefore, another test was performed at 120 °C during 6 h (entry 6). Compared to 120 °C/2 h (Table 3, entry 5), the conversion was expectedly improved (90% vs 44%) and the yield of benzoic acid was higher (19% vs 7.5%). However, the yield of phenol was not improved significantly and the carbon balance fell to 55% (vs 96%) meaning there is a higher carbon loss due to side reactions.

To conclude, ideally **A1**_HH_ cleavage tests should be performed at 120 °C during 2 h (Table 3, entry 5) since a reasonable compromise can be reached between the **A1**_HH_ conversion, the yields of PhOH, PhCHO and PhCOOH and a correct mass balance in these conditions.

### 3.4. Lignin Aerobic Cleavage: Preliminary Tests

**Figure 9:**
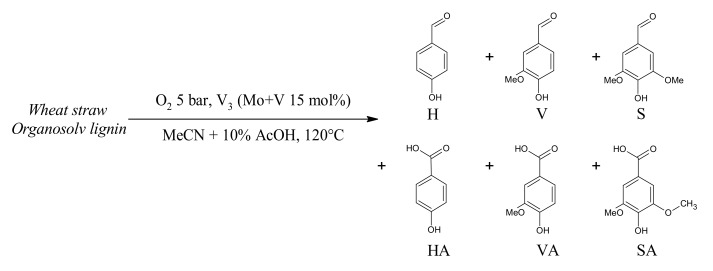
Aerobic oxidative cleavage of lignin (WSLp) catalyzed by H_6_PV_3_.

The catalytic tests on lignin (WSLp) were performed directly under 5 bars of O_2_ (measured at R.T.) at 120° during either 2 or 6 h (Figure 9) like in the case of **A1**_HH_.

The expected aromatic aldehydes noted H, V and S could be detected by GC-MS (see Table S3). However, the expected phenolic acids were barely evidenced by GC-MS. Since those acids are difficult to detect with apolar GC columns and are not very stable at high temperature [48], the reaction mixture had to be treated overnight by an excess of N,O-Bis(trimethylsilyl)trifluoroacetamide (BSTFA) for quantification of the trimethylsilyl derivatives by GC-MS. Alcohols and acids were both derivatized. The reaction medium was also injected without silylation to check the presence of by-products such as 2,6-DiMethoxyBenzoQuinone (2,6-DMBQ) or acetosyringone. The retention times of the main derivatized oxidation products identified are given in Table S3. The GC-MS analyses of the reaction media before silylation also emphasized the formation of p-quinones derivatives such as 2,6-DMBQ which are products of the C(Ar)-C cleavage, as observed by Evtuguin et al. [27]. Such a reaction would take place due to the presence of phenolic functions since these quinones were observed only as minor by-product in the case of the oxidative cleavage of the non-phenolic models, especially for **A1**_HH_. Acetovanillone was produced too. This may be originated from either an oxidative or a non-oxidative C-O cleavage, accelerated by the acidity brought by the catalyst [25,27].

The yields of the targeted soluble products, determined by GC-MS (calibration curves given on Figure S8) were rather low with a sum for H, HA, V, VA, S and SA (expressed in mol%) lower than 3% in the best case (120 °C, 6 h, Table 4, entry 3). The catalyst turned out to be necessary for lignin aerobic cleavage (entries 1 vs. 2) since V, VA and HA monomers were not detected after 2 h at 120 °C in the absence of V_3_. Moreover, the extent of lignin dissolution (9–10 wt%) was rather unchanged by the catalyst. At 120 °C, a longer reaction time had a positive impact on the yield of cleavage products (Table 4, entries 3 vs. 1).

## 4. Discussion

### 4.1. H_6_PV_3_ Synthesis

The targeted x value (3) could be almost obtained by following the hydrothermal synthesis (experimental ×: 2.7). Yet, compared to Reference 31 (applied for V_1_ synthesis), a longer attack (6.5 h instead of 3 h) and more diluted conditions were needed to dissolve the oxides. However, part of the oxides did not dissolve and had to be eliminated by filtration despite the use of more diluted conditions and long heating time. The solid residue was found to be richer in vanadium (w_Mo_ = 23 wt.%, w_v_ = 38 wt.%) than the initial mixture of oxides. A reflux of 24 h was tested to enhance the dissolution but the amount of residual oxide did not decrease significantly meaning that a longer attack is not efficient. Yet, inclusion of vanadium was better compared to that reported by GRATE et al. [36] (V_2.4_ instead of V_3_) despite a much longer heating step (6.5 h vs 168 h). It is known that H_6_PV_3_ has a rather low thermal instability and stability to the hydrolysis of VO_2_^+^ generated from the parent H_6_PV_3_ [30]. As a result, the long heating duration applied by Grate et al. may have favored H_6_PV_3_ destruction rather than the dissolution of the starting oxides.

### 4.2. Evolution of the Catalyst

**Figure 10:**
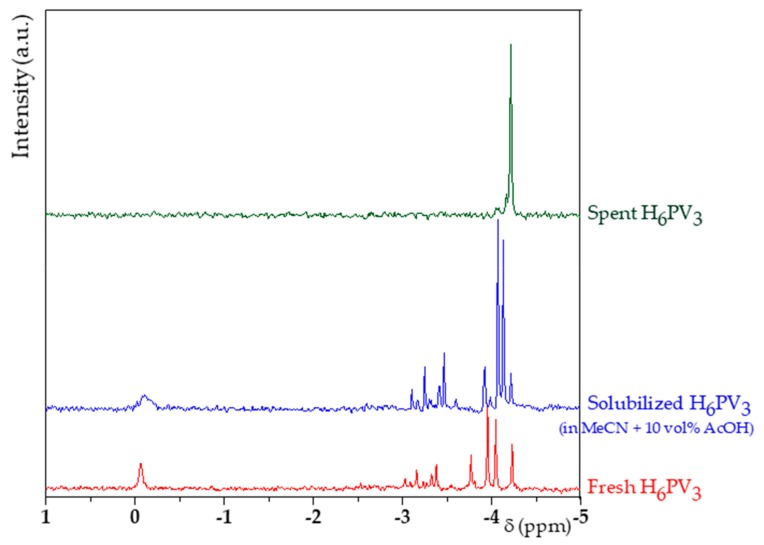
^31^P NMR spectra of the fresh and spent H_6_PV_3_ catalysts (catalyst 30 mg in D_2_O-H_2_O 50:50 500 µL + dioxane 7.5 µL).

After **K1**_HH_ oxidation (Table 2, entry 6), the catalyst was recovered and analyzed by ^31^P NMR showing only one peak at −4.2 ppm (Figure 10) instead of several ones as shown in Figure 3. The latter, measured at pH = 1, could be attributed to the phosphomolybdate anion. Having checked, in parallel, that the Keggin-type structure was preserved when only heating H_6_PV_3_ in MeCN - 10 vol% AcOH (80 °C), we may reasonably suggest that H_6_PV_3_ was not stable upon the reaction conditions, being transformed into a mixture of phosphomolybdate anion and, a priori, vanadyl (VO^2+^) or pervanadyl (VO_2_^+^) counter-cations. Indeed, it has been reported by Neumann and Khenkin that H_3+x_PV_x_ compounds may lose a vanadyl cation when they are reduced by the substrate (here, H-Su) as the result of the increased distance between the heteroatom P and vanadium [49]. Such process is summarized in Equation (1) for x = 3.

(1) $${\left[{\mathrm{PMo}}_{9}V^{+V}{}_{3}O_{40}\right]}^{6-}+H-\mathrm{Su}={\left[{\mathrm{PMo}}_{9}V^{+V}{}_{2}O_{39}\right]}^{9-}+V^{+IV}O^{2+}+H^{+}+{\mathrm{Su}}^{.}.$$

Sometimes, H_3+x_PV_x_ are also considered as the precursors of in situ-formed pervanadyl cations that would be the true catalysts as suggested by Bregeault et al. [50] and El Amrani et al. [51], characterized by a higher redox potential than parent H_3+x_PV_x_ [29] and therefore may be more effective for oxidation, including aerobic cleavage. Such ejection is accompanied by the formation of an unstable lacunary polyoxometalate (POM) that generates a phosphomolybdate anion. Such reaction, favored by the acidity can be described by the equation Equation (2) [28] for x = 3:

(2) $$H_{6}\left[{\mathrm{PMo}}_{9}V^{+V}{}_{3}O_{40}\right]+H^{+}=H_{5}\left[{\mathrm{PMo}}_{9}V^{+V}{}_{2}O_{37}\right]+V^{+V}O_{2}^{+}+H_{2}O$$

### 4.3. **K****1**_HH_ and **A****1**_HH_ Cleavage

To get insight on the role of acetic acid, the O_2_ solubility in binary acetonitrile-acetic acid was also estimated using the equations established by Sato et al. [44] that relate the solubility of gases in solvents mixtures to their solubility in the corresponding pure solvents using Hansen parameters (details in the Supporting Information).

By analogy with El Aakel et al. [14], acetic acid and methanol were tested as solvents for the C-C cleavage of **K1**_HH_. In their work, methanol proved to be the most adequate solvent in the case of benzoin (Ph-CO-CH(OH)-Ph, r. T., atm. O_2_), which is not the case here for **K1**_HH_. Likewise, benzoin (H_6_PV_3_, r. T.) [14] or cyclohexanone (V_2_, 70 °C) [52], the active site in **K1**_HH_ would be C^β^ (see Figure 1). However, the C-H activation of C^β^ would be easier in **K1**_HH_ than in cyclohexanone as a result of the presence of an electro-donating phenoxy group stabilizing the transient radical.

We hypothesize that the enhancement of the yields of cleavage products in presence of acetic acid is related to a better solubility of O_2_. Indeed, with trifluoroacetic acid (TFA, 3.6 vol.%), which is a much stronger acid than acetic acid [53] but which has a similar affinity to molecular oxygen (solubility in TFA: 8.1 mM [54] vs 7.6 mM in acetic acid, see Table 2), no improvement of the C-C cleavage products was observed. The combined yield of PhCHO and PhCOOH was 38% vs 63% in presence of AcOH 10 vol.% meaning that acidity has a lower impact than O_2_ solubility. However, it can be noticed from Figure S9, that the addition of 10 vol.% of acetic acid in acetonitrile leads to a raise of molecular oxygen solubility of 11% only which means that acetic acid would have another role. Indeed, we suggest that, in presence of acetic acid, the C-C cleavage would be accelerated by a nucleophilic substitution reaction between the phenoxy group of **K1**_HH_ and acetic acid leading to 2-acetoxyacetophenone observed by GC-MS. Indeed, such reaction can take place even without H_6_PV_3_ (Table 2, entry 9) in presence of 10 vol.% of acetic acid as shown by the large excess of phenol. The substitution reaction of -OPh by -OAc may also make the C-C oxidative cleavage easier due to a decrease of the steric hindrance. A last role of acetic acid could originate from its known ability to stabilize the pervanadyl cation VO_2_^+^ as proposed by MA et al. [23] in their work dealing with the aerobic oxidation of **A1**_HH_ catalyzed by VO(acac)_2_ in MeOH-AcOH 8 vol%. Indeed, acetic acid would contribute to an easier regeneration of the active form of the catalyst through aerobic oxidation. However, the pH of the reaction media in our work should be lower compared to that measured by Ma et al. since H_6_PV_3_ and H_3_PMo_12_, obtained by the decomposition of H_6_PV_3_, are strong acids. Acetic acid being actually a weak acid in acetonitrile [53], the presence of H_3+x_PV_x_ or of H_3_PMo_12_ should inhibit acetic acid deprotonation.

The present contribution dealt with the cleavage of two non-phenolic models **K1**_HH_ and **A1**_HH_. Our results with the latter can be compared to those of Evtuguin et al. [27] who used a H_8_PV_5_ catalyst (480 mol% of Mo + V, O_2_ 5 bar, acetone-water 70/30 v/v, 90 °C, 20 min) in the presence of another alcohol with electro-donating methoxy groups. In our case, it is noteworthy that, despite the absence of any activating groups and the choice of a lower metal loading (15 mol% vs 480 mol%), a higher conversion 44% ((120 °C, 2 h), MeCN - 10 vol% AcOH), **vs** 20% (90 °C, 20 min, acetone-water) and a higher selectivity for C-C cleavage (24% vs 18%) were obtained. However, Evtuguin et al. used acetone-water 70/30 v/v as a solvent which is less prone to solubilize O_2_ (1.2 mM [44,45]) compared to MeCN - 10 vol% AcOH (2.6 mM). Such result confirms that molecular oxygen transfer has a critical impact on aerobic cleavage and a reaction time of 20 min is too short to observe high yields of C-C cleavage.

### 4.4. Mechanism Proposal

In order to establish a plausible mechanism for **K1**_HH_ cleavage, we also tried to identify the minor products by using GC-MS analysis. The different cleavage products detected are listed in Table S2 depending on their origin (C-O cleavage (Table S2a), C-C cleavage (Table S2b)). p-Quinone (from phenol oxidation) as well as phenylglyoxylic acid and 2-acetoxyacetophenone (from the acetolysis of **K1**_HH_, Table S2a) were identified. In addition, N-acetylbenzamide (Table S2b) could be detected. This amide would result from the reaction of benzoic acid with acetamide, the latter being produced by the hydrolysis of acetonitrile. Phenyl formate was also observed (Table S2b), meaning that formic acid maybe produced during the reaction.

**Figure 11:**
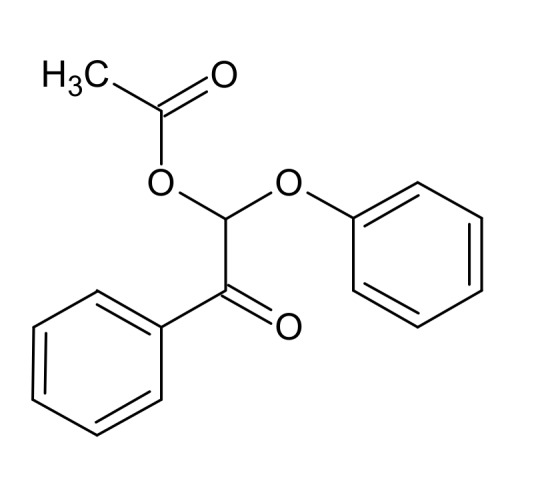
Hypothetic structure of the intermediate X.

The analysis of the undiluted sample allowed us to detect 1-(2-hydroxyphenyl)-2-phenoxyethanone and its isomer 2-(2-hydroxyphenyl)-1-phenoxyethanone (Table S2c). Both of them would be produced by the hydroxylation of the aromatic rings of **K1**_HH_ by HO^.^ radicals. Another compound (X) would be 2-phenoxy-2-acetoxy-1-phenylethanone (Figure 11 and Figure S10, Table S4).

**Figure 12:**
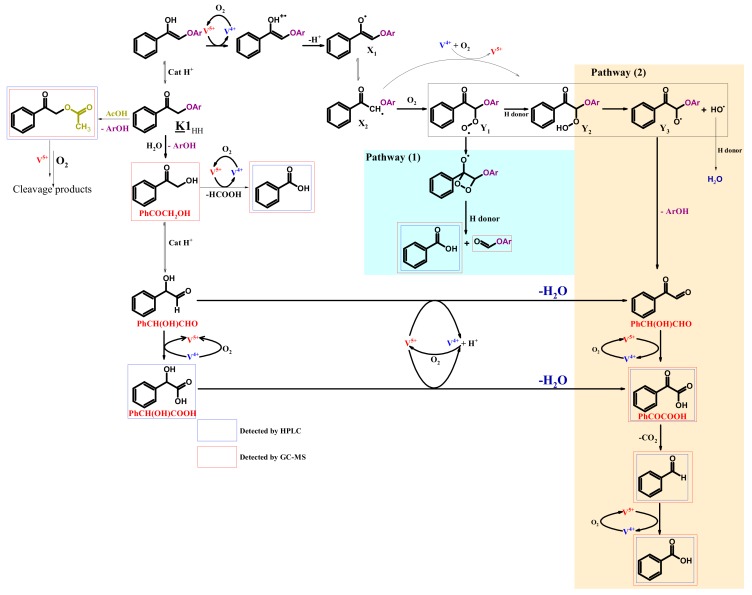
Mechanism proposal for the H_6_PV_3_ catalyzed aerobic cleavage of **K1**_HH_.

A mechanistic pathway explaining the formation of the main products, other compounds being either intermediates or products from dead-end routes is proposed in Figure 12.

Hence, benzoic acid would be generated directly from **K1**_HH_ through a direct C-C cleavage via a peroxyl radical intermediate (pathway (1)) [55], or through the C-O cleavage of a free radical intermediate **Y_3_** (pathway (2)). Y_3_ could have reacted with acetic acid to form **X** (supposed from GC-MS data).

By analogy with cyclohexanone, studied by Cavani et al. [52], **K1**_HH_ may be also tautomerized into its corresponding enol ether (Figure 12) as a result of an acid-catalyzed process involving H_3+x_PV_x_. One electron oxidation of the enol by vanadium (V) can be envisaged [13,56] affording **X_1_** and **X_2_** radicals. Then, **X_2_** may react with molecular oxygen, giving rise to a hydroperoxyl radical, Y_1_ [49]. The latter would react with a hydrogen donor, for example, the substrate itself [52] affording a hydroperoxide **Y_2_**. It has to be noted that **Y_2_** could also be produced by the reaction of O_2_^.−^ with the **X_2_** radical followed by a protonation step. Such superoxide anions would be formed in-situ by the one-electron oxidation of vanadium (+IV) by molecular oxygen [57]. Homolytic cleavage of the O-O bond in **Y_2_** would occur afterwards, leading to **Y_3_** then to phenylglyoxal and a phenoxy radical that can be reduced into phenol. Further oxidation of phenylglyoxal may give an α-keto carboxylic acid, phenylglyoxylic acid that can be decarboxylated, thus producing benzaldehyde and CO_2_. Phenylglyoxal may also be formed from 2-hydroxyacetophenone, the hydrolysis product of **K1**_HH_ of which only traces were observed due to its high reactivity compared to **K1_HH_** as the result of the presence of the OH group [14,50].

Hydrolysis was shown indirectly by the presence of mandelic acid (PhCH(OH)COOH) obtained from the isomerization of 2-hydroxyacetophenone (PhCOCH_2_OH) into mandelaldehyde (PhCH(OH)CHO) and then aerobic oxidation [58]. Benzaldehyde cannot be formed through the pathway (1), that is, cleavage of the C-C bond into phenol and phenyl formate, which means that the pathway (2) and also the oxidation via the hydrolysis product (2-hydroxyacetophenone) take place. Yet, the formation of substantial amounts of phenyl formate shows that the oxidative cleavage occurs through pathway (1) too. Let us notice that i) the oxidation through pathway (1) and ii) an initial hydrolysis of **K1**_HH_ and cleavage into PhCHO and CO_2_ consumes at least one equivalent of molecular oxygen whereas iii) the oxidation into PhOH and PhCHO through pathway (2) consumes at least 1.5 equivalents of molecular oxygen and one equivalent of carbon dioxide is emitted. So, the observed oxygen consumption (1.5 mol/converted **K1**_HH_) is adequate to this mechanism.

**Figure 13:**
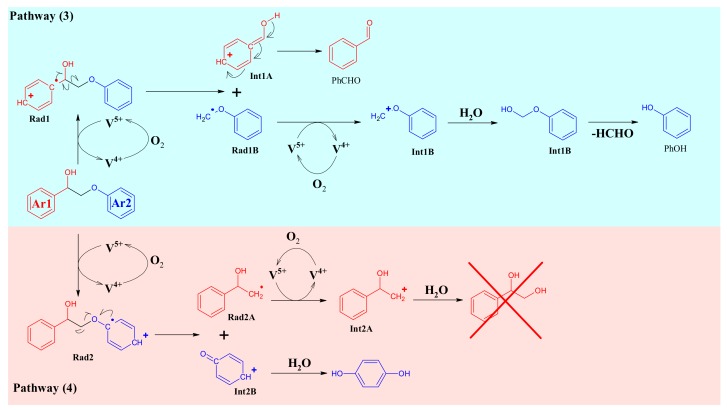
Mechanisms suggested for the direct cleavage of **A1**_HH_ inspired by EVTUGUIN et al.

A similar search for the by-products was done also in the case of **A1**_HH_ leading to a classification of the cleavage products arising either from C-O and C-C cleavage or products from side reactions (Table S2a–c). By analogy with **K1**_HH_, phenyl formate (C-C cleavage) and acetate (C-O cleavage), 2-acetoxyacetophenone (acetolysis) and phenyl benzoate were observed as minor products from **A1**_HH_ cleavage but some other compounds characteristic of **A1**_HH_ cleavage, such as phenylacetaldehyde and hydroxystilbenes were also evidenced. A first possible mechanism for **A1**_HH_ cleavage would involve the oxidation of **A1**_HH_ into **K1**_HH_, then the different pathways described in Figure 12. Indeed, **K1**_HH_ was detected as a main product and 2-phenoxyacetophenone, as well as 2-phenoxy-2-acetoxy-1phenylethanone were also detected. However, the PhCHO/PhCOOH ratio in **A1**_HH_ cleavage was shown to be higher than in **K1**_HH_ cleavage which could be related to the involvement of another mechanism (Figure 13) inspired by Evtuguin et al. [27].

Two pathways were suggested—the first one (pathway (3)) involves a monoelectronic oxidation of the **Ar1** moiety by the catalyst into a radial cation, Rad1. This activates the substrate for C-C cleavage into the **Int1A** cation and the radical, Rad1B. Then, **Int1A** would be deprotonated giving rise to benzaldehyde while **Rad1B** would be further oxidized (monoelectronic transfer) into the **Int1B** cation whose hydration leads to a formaldehyde hemiacetal. Finally, this one would be cleaved into phenol and formaldehyde. The second mechanism (pathway (4)) involves a monoelectronic oxidation of the **Ar2** moiety into the radical cation, **Rad2**. Then, C-O cleavage in **Rad2** would take place, affording the **Rad2A** radical and the **Int2B** cation. **Int2B** may be hydrated into hydroquinone while **Rad2A** is further oxidized (monoelectronic transfer) into the **Int2A** cation that is hydrated into styrenediol.

**Figure 14:**
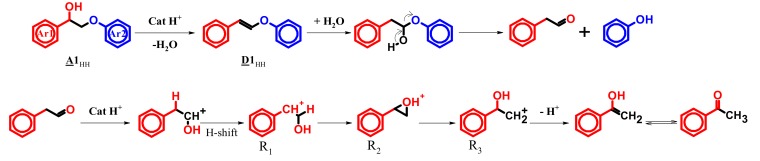
Suggested mechanisms for the formation of phenylacetaldehyde and acetophenone.

At first sight, styrene diol and hydroquinone were not detected by GC-MS, which would invalidate the pathway (4). So, the magnitude order of the PhCHO/PhCOOH ratio would be explained by **A1**_HH_ oxidation according pathway (3) only. However, the formation of phenylacetaldehyde and acetophenone is not taken into account in Figure 12 and Figure 13. A priori, such products are generated by a non-oxidative C-O cleavage (Figure 14). Their common precursor would be the dehydration product of **A1**_HH_, that is, **D1**_HH_. Hence, **D1**_HH_ would be rehydrated then into an unstable hemiacetal whose C-O cleavage gives rise to phenol and phenylacetaldehyde. The latter one may also isomerize into acetophenone in acidic media (Figure 14). Basically, such reaction would proceed through an acid-catalyzed H-shift [59], boosted by the enhanced stability of the **R_1_** carbocation which in turn gives rise to a protonated epoxide **R_2_** as a transient species. The ring is then opened, affording **R_3_** and, after deprotonation and tautomerization, acetophenone. It has to be noticed that phenylacetaldehyde polymerizes easily, even at room temperature [60], that may be the reason why this aldehyde could not be detected by HPLC and GC-MS. As a consequence, it has to be emphasized that, on the contrary to esterification, the dehydration pathway cannot be considered at all as a dead-end route. However, it essentially gives rise to a non-oxidative C-O cleavage with a risk of polymerization.

**Figure 15:**
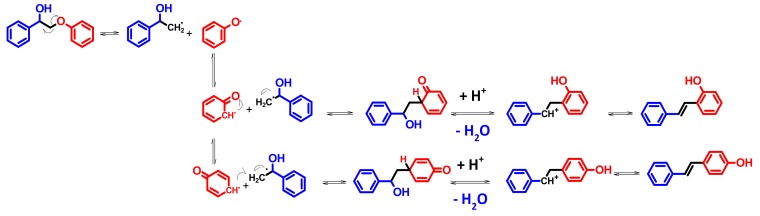
Suggested mechanisms for the formation of hydroxystilbenes.

Finally, hydroxystilbenes were shown to be minor side products of the oxidative condensation. Knowing that the C-O bond is the weakest linkage in lignin [61] and in its models too, we can suggest a homolytic cleavage (as depicted in Figure 15) affording one O- and one C- centered radicals. Then, inspired by lignification process [62] and knowing that repolymerized lignin contains a higher amount of C-C bonds compared to native lignin [61], the second step on the mechanism would be the isomerization of the phenoxy radical into a new C-centered radical and its homolytic coupling with the other one, previously formed. The hydroxystilbenes would be finally obtained through a dehydration step.

### 4.5. Extension to Lignin

It is well-known that lignin models have not really the reactivity of lignin. Therefore, the extension to lignin had to be addressed. The yields of C-C cleavage products from the Organosolv lignin were really low as shown in Table 4, even lower compared to **A1**_HH_ cleavage. Similarly to the **A1**_HH_ cleavage, the reason would be the esterification of the alcohol functions during the reaction. To get insight on the fate of the hydroxyl groups, a ^31^P NMR analysis of phosphorylated lignin (reaction given in Figure S11) samples before and after oxidation was performed. An example of spectrum of lignin before oxidation is given in Figure S12a. The signals related to lignin are given in Figure S12b and their attribution in Table S5. It can be noticed that the fractions of phenolic OH functions (21 vs 40%) decreased in the run performed without the H_6_PV_3_ catalyst (Table 4, entry 3) meaning that these functions were the most reactive, but for oxidative condensation since almost no C-C cleavage products was observed even after silylation. The drop of phenolic moieties content took place at a lesser extent in presence of catalyst meaning that a higher amount of aliphatic functions may be chemically transformed, which is compatible to a higher yield of C-C cleavage reactions as observed by GC-MS. In addition, we observed that oxidized lignin (120 °C, 2 h) either in the presence or without H_6_PV_3_ is much less soluble in dimethyl sulfoxide (DMSO) (2 g/L vs >20 g/L for lignin before oxidation) and in CDCl_3_-pyridine 1-1.6 v/v (after phosphorylation). The oxidized lignin (O_2_ 5 bar, Mo + V 15 mol%, 120 °C, 2 h) was also tested for aerobic cleavage in the same operative conditions and showed to be resistant to cleavage and no dissolution was observed. This is due to the formation of more recalcitrant C-C bonds at the expense of C-O linkages [61] through condensation reactions as observed for **A1**_HH_ cleavage but at a much higher extent because of the presence of phenolic groups [61,63].

It was noticed that 9 wt% of lignin was dissolved either with or without catalyst. The solubility cannot be due to the formation of monoaromatics only but also soluble oligomers formation from oxidative cleavage or acid-catalyzed C-O cleavage. The distribution of H (46%–50%), G (36%–38%) and S (12%–16%) monoaromatic compounds was rather unchanged, meaning that the different monomers had similar reactivities.

## 5. Conclusions

A Keggin-type molybdovanadophosphoric acid with the following mean formulae H_5.7_[PMo_9.3_V_2.7_O_40_] (H_6_PV_3_) was easily synthesized (79% yield) through a hydrothermal procedure and characterized by XRD, ^31^P NMR in D_2_O-H_2_O 50:50, ICP and TGA. The addition of 10 vol% of acetic acid to an oxidation resistant solvent, such as acetonitrile, was shown to be beneficial for the ketone cleavage mainly as the result of an enhanced molecular oxygen solubility. In the optimized conditions (82 °C, 24 h), the conversion of the ketone **K1**_HH_ reached 72% and the yield of PhOH, so as the combined yield of PhCHO and PhCOOH were around 55%. Nevertheless, **K1**_HH_ is quite far from lignin compared to the alcohol model **A1**_HH_. The latter one proved to be more resistant and the O_2_ pressure had to be increased. Besides, the acetate ester of **A1**_HH_, formed as a main by-product, turned out to be even more resistant than **A1**_HH_ which indicates that esterification has to be mitigated. A tentative of optimization of the O_2_ pressure and of the reaction temperature was carried out for **A1**_HH_ cleavage. Yet, at high pressure and temperature, the carbon balance tended to be low, implying that side reactions such as hydroxystilbenes formation through oxidative condensation and tar formation are involved. As a result, a temperature of 120 °C, a molecular oxygen pressure of 5 bar and a duration of 2 h were the most adequate parameters.

From the mechanistic studies, it was shown that, in **K1**_HH_, the active site is the CH_2_ group and that the C-C cleavage may proceed either through hydrolysis of **K1**_HH_ or through the formation of a hydroperoxyl radical. The latter one may be cleaved directly into phenyl formate and benzoic acid or transformed into a radical from hydroxylated **K1**_HH_ evoluting to phenol and phenylglyoxal that is further oxidized into phenylglyoxylic acid whose decarboxylation generates benzaldehyde. **A1**_HH_ cleavage may be direct or proceed through alcohol oxidation into the ketone **K1**_HH_. Acidic C-O cleavage was evidenced as a side reaction.

Since the alcohol is more resistant to cleavage and the benzylic alcohol moiety (carbon C^α^) is an active site for both dead-end oxidative condensation and esterification, a stepwise procedure may be a more suitable strategy [64] to cut down these side reactions. It was used not only for oxidative cleavage but also for reductive and acidic cleavage of lignin models [64,65,66,67]. For example, the first step would consist in oxidizing the benzylic alcohol into a ketone in presence of a catalytic system involving an oxidant such as TEMPO ((2,2,6,6-Tetramethylpiperidin-1-yl)oxidanyl) [68] and then, ketone cleavage should be performed in atmospheric conditions.

## Figures and Tables

**Table 1:** Cell parameters of H_6_PV_3_ catalysts (vs reference H_3_PMo_12_) calculated on Fullprof^®^ by the RIETVELD method.

Material	a (Å)	b (Å)	c (Å)	α (°)	β (°)	γ (°)	R_P_ (%)	Chi_2_ (%)
V_3_	14.219	14.410	13.616	112.54	110.13	60.13	15.1	13.1
H_3_PMo_12_ (JCPDS 00-043-0317)	14.100	14.130	13.550	112.10	109.80	60.70	-	-

**Table 2:** Influence of acetic acid content on the activity of H_6_PV_3_ on the **K1**_HH_ aerobic oxidative cleavage.

Entry	z (vol%)	Mo + V Loading (mol%)	Conv. (%)	Yield (%)			O_2_ sol. (%) ^a^	CB (%)	O_2_ Consumption ^b^
				PhOH	PhCHO	PhCOOH			
**1** ^c^	0	15	14	8.1	0	4	2.1 ^d^	91	n. i.
**2**	0	15	48	32	2.5	19	2.3 ^d^	76	0.9
**3**	100	15	100	80	0	80	7.8 ^d^	74	0
**4**	2.5	15	59	34	17	28	2.36	78	2.0
**5**	5	15	68	47	20	38	2.42	81	*2.9* ^e^
**6**	10	15	72	55	12	44	2.56	80	1.5
**7** ^f^	10	15	44	24	5.6	24	2.56	81	1.5
**8**	20	15	80	67	0	47	2.85	72	1.4
**9** ^g^	10	0	39	35	11	0.9	2.56	82	1.3
**10**	10	7	43	36	8	24	2.56	88	1.4
**11**	10	36	88	47	2.2	50	2.56	58	1.1

**K1_HH_** 100 mM, atm. O_2_, H_6_PV_3_ (Mo + V 15 mol%), MeCN − z vol% AcOH, 82 °C, 24 h; n. d. = not detected. ^a^ The solubility in mixed solvents was estimated using equations from Ref 44; ^b^ Normalized to conversion; ^c^ Methanol (65 °C); ^d^ Obtained from Refs. [45,46]; ^e^ Due to experimental error; ^f^ T = 65 °C; ^g^ H2SO4 (5 µL) was used instead of V3.

**Table 3:** Activity of H_6_PV_3_ catalyst for **A1**_HH_ cleavage.

Entry	T (°C)	P (bar)	t (h)	Conv. (%)	Yield (%)						CB (%)
					PhOH	PhCHO	PhCOOH	K1_HH_	Est^α^A1_HH_	D1_HH_	
**1**	80	1	24	35	4.6	2.0	2.1	17	9	13	108
**2** ^a^	80	1	24	9.1	1.9	2.0	tr	tr	0	5.2	98
**3**	80	5	24	54	16	7.1	9.8	10	18	21	118
**4**	80	5	6	33	3.7	2.9	3.5	8.7	9.9	10	99
**5**	120	5	2	44	7.1	6.4	7.5	2.7	11	16	96
**6**	120	5	6	90	7.6	2.6	19	6.7	12	12	55

**A1**_HH_ 100 mM, H_6_PV_3_ (Mo + V 15 mol%), MeCN - 10 vol% AcOH; ^a^ Without catalyst (in presence of H_2_SO_4_ 6.2 mol%).

**Table 4:** H_6_PV_3_ catalyzed lignin aerobic cleavage.

Entry	t (h)	Liquid Phase (GC-MS after Silylation)						Residual Lignin (^31^P NMR)			
		Yield of Monoaromatics (mol%) ^b^						Function Ratio	Monoaromatic Type Proportions ^b^		
		Units H		Units G		Units S		Aliph./ArOH/COOH	H	G	S
		H	HA	V	VA	S	SA				
1	2	0.4	0.1	1.2	0.3	0.2	0	40/30/30	16	36	48
2 ^a^	2	0	0	0.1	0	0.2	0	39/21/40	12	38	50
3	6	0.6	0.3	0.7	0.4	0.3	0	n. d.	n. d.		
WSL_p_	-	-	-	-	-	-	-	36/40/24	16	38	46

WSL_p_ 0.85 g (equivalent to 5 mmol of monoaromatic compounds); H_6_PV_3_ (Mo + V 15.9 mol% vs the estimated amount of dimeric units), 120 °C, O_2_ 5 bar; ^a^ Without any catalyst, ^b^ Calculated from ^31^P NMR data.

## Supplementary Materials

The following are available online at https://www.mdpi.com/1996-1944/13/4/812/s1, synthesis procedure of **K****1**_HH_, **A****1**_HH_ and **Est**^α^**A****1**_HH_, a general HPLC profile of the reaction media (Figure S1), the calibration curves of PhOH, PhCHO, PhCOOH, **K****1**_HH_, **A****1**_HH_ and **Est**^α^**A****1**_HH_ (Figure S2), the list of products identified by HPLC (Table S1) and by GC-MS (Tables S2a–c), XRD profiles of H_6_PV_3_ vs starting MoO_3_ and V_2_O_5_ and reference H_3_PMo_12_, Figure S3) and a Rietveld refinement (Figure S4), graphical relaxation delay of H_6_PV_3_ (FigureS5 and S6), graphical calculation of the kinetic order to **K****1**_HH_ and **A****1**_HH_ (Figure S7), the calibration curves of phenolic aldehydes and acids from C-C cleavage of lignin (Figure S8), details on the calculation of oxygen solubility in binary acetonitrile and acetic acid (Figure S9), list of Organosolv lignin aerobic C-C cleavage identified by GC-MS (Table S3), identification of the structure of the intermediate X (Table S4 and Figure S10) and ^31^P NMR analysis of phosphorylated lignin (reaction detailed in Figure S11 and spectrum in Figure S12a and S12b, identification list on Table S5).
