# Supplementary material for: Vanadium-Substituted Phosphomolybdic Acids for the Aerobic Cleavage of Lignin Models—Mechanistic Aspect and Extension to Lignin
Source: Materials (Basel). 2020 Feb 11;13(4):812. doi: 10.3390/ma13040812 (PMC7079653; doi:10.3390/ma13040812)
Supplement: Supplementary file 1 [file materials-13-00812-s001.pdf]

materials

MDPI

Supporting information

Vanadium-Substituted Phosphomolybdic Acids for the Aerobic Cleavage of Lignin Models—Mechanistic Aspect and Extension to Lignin

Louay Al-Hussaini [1,2], Franck Launay [1,]* and Elena Galvez [2]

Synthesis of **K1**$_{HH}$, **A1**$_{HH}$ and **Est$^{\alpha}$A1**$_{HH}$ [S1,S2]

2-bromoacetophenone (16.85 g, 84 mmol) and an excess of phenol (9.56 g, 101 mmol) were dissolved in 200 mL of acetone. Added to the solution was 20 g of potassium carbonate, used as a catalyst and as an acid (HBr) trap. The mixture may take a pink coloration due to the formation of phenolate anions that disappears with time. Reflux is needed during 6 h to get **K1**$_{HH}$. Initially pale yellow, the coloration became yellow and then orange, meaning that all the brominated reactant is consumed and phenol begins to be oxidized by air. The reaction was monitored by TLC using cyclohexane/diethylether 80/20 as the eluent. After filtration and acetone evaporation, **K**1$_{HH}$ was recrystallized in a minimum amount of heated (65–70 °C) absolute ethanol. **K1**$_{HH}$ was then recovered by filtration and dried by pressing. The formation of the ether bond was checked by Fourier-transform–infrared (FT-IR) by the presence of a band at 1240 cm^{-1}. **K1**$_{HH}$ was characterized more deeply by ^{1}H nuclear magnetic resonance (NMR) according to Ref S1.

^{1}H NMR (CD_3CN, 300 MHz): 8.05 (m, 2H), 7.66 (m, 1H), 7.54 (m, 2H), 7.33 (m, 2H), 7.00 (m, 3H), 5.31 (s, 2H).

This above-mentioned procedure was repeated several times at different scales affording yields of **K1**$_{HH}$ of c.a. 80%.

To get **A1**$_{HH}$, **K1**$_{HH}$ (4.98 g, 23 mmol) was solubilized in 52 mL of THF and 13 mL of H_2O and reduced by $NaBH_4$ (1.32 g, 35 mmol) for 5 h. The reaction was monitored by TLC using a binary (80/20) cyclohexane/diethylether mixture. Afterwards, the reaction was quenched by 120 mL of saturated NH_4Cl (added slowly because of H_2 emission) and then diluted by 120 mL of water. Later, **A1**$_{HH}$ was extracted by diethyl ether (250 + 120 mL). After washing the diethylether fractions by brine (120 mL) and drying by anhydrous $MgSO_4$, the solvent was evaporated to get **A1**$_{HH}$ as a white solid with a yield of 90% on average. FT-IR spectroscopy enabled to check the disappearance of the strong signal at 1700 cm^{-1} due to the carbonyl group reduction. **A1**$_{HH}$ was deeply characterized by ^{1}H NMR (according to Ref S1).

^{1}H NMR (CD_3CN, 300 MHz): 7.39 (m, 7H), 6.97 (m, 3H), 5.04 (dd, 1H), 4.13 (dd, H), 4.05 (dd, 1H).

A1$_{HH}$ was esterified by acetic anhydride in pyridine giving rise to **Est$^{\alpha}$A1**$_{HH}$. Hence, 505 mg of **A1**$_{HH}$, were dissolved in 5 mL of pyridine in a round-bottom flask and 5 mL of acetic anhydride were dropped. The resulting mixture was stirred during 17 h and the total consumption of **A1**$_{HH}$ monitored by TLC with an 80/20 cyclohexane/diethylether eluent. Excess of anhydride was quenched by 20 mL of methanol (Be careful! This quenching reaction is very exothermic and the mixture may boil). After cooling, the solvent was evaporated. Then, the concentrated residue was dissolved again in methanol (≈5 mL) and the mixture was dropped in a 20 mL volumetric flask. The first flask was washed by methanol. The washing fraction was poured also in the volumetric flask. Then, the volume was completed to 20 mL. The solution was injected by high performance liquid chromatography (HPLC) to check the retention time. Afterwards, the solvent was evaporated then to eliminate the most possible the pyridine. The obtained ester noted **Est$^{\alpha}$A1**$_{HH}$ was then purified on a chromatographic column (eluent: pentane/diethylether 1/1). After evaporation of the solvent, 489 mg (yield 97%) of pure **Est**$^{\alpha}$**A1**$_{HH}$ were collected as a colorless oil and characterized according a predicted spectrum.

RMN ^{1}H ($CDCl_3$, 300 MHz): 7.37 (m, 7H), 6.96 (m, 3H), 6.18 (dd, 1H), 4.30 (dd, H), 4.18 (dd, 1H), 2.14 (s, 3H).

HPLC

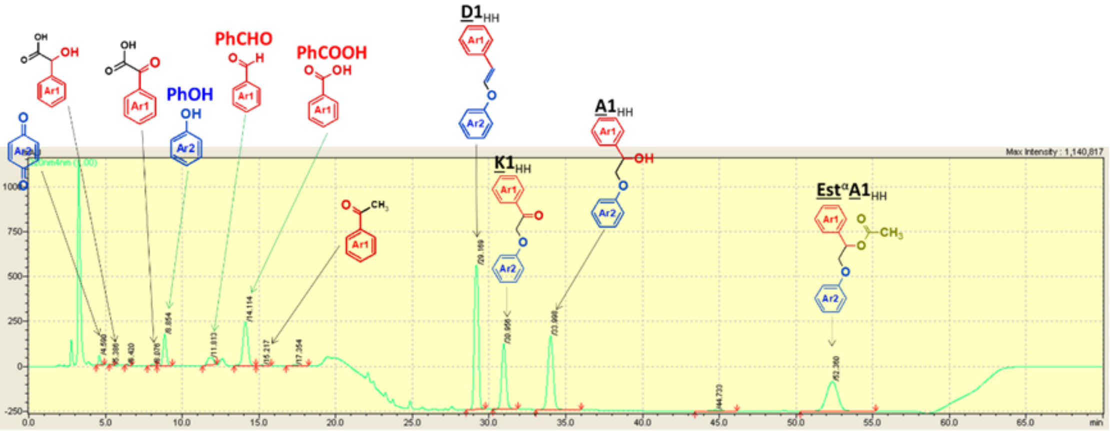

Figure S1. Typical high performance liquid chormatography (HPLC) profile of **A1**HH cleavage in presence of V_3 (220 nm).

HPLC

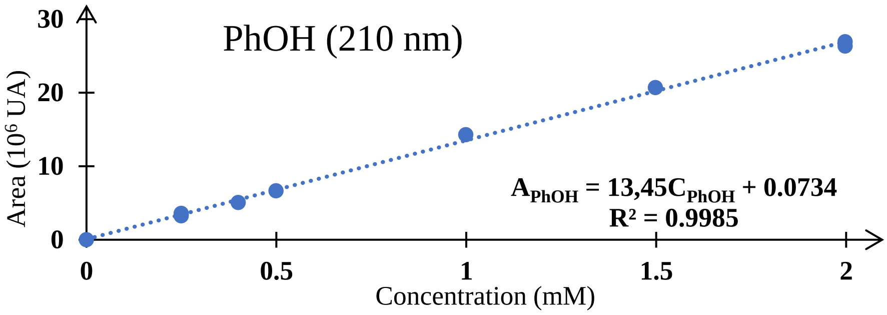

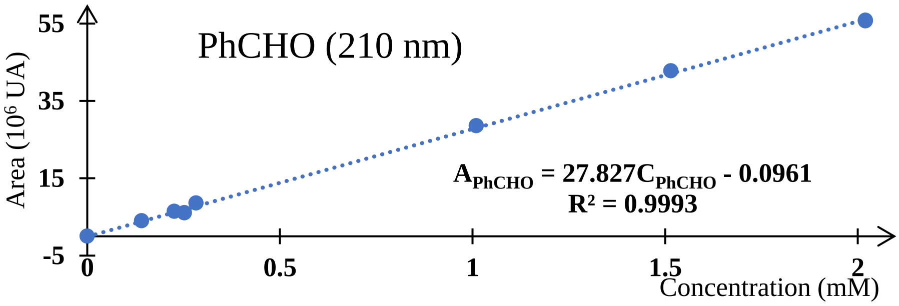

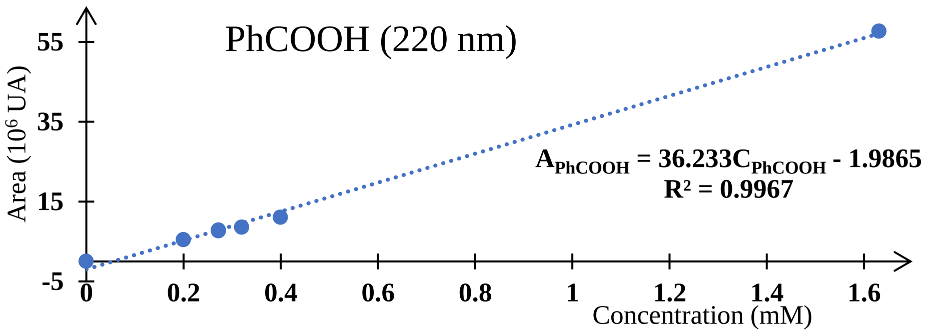

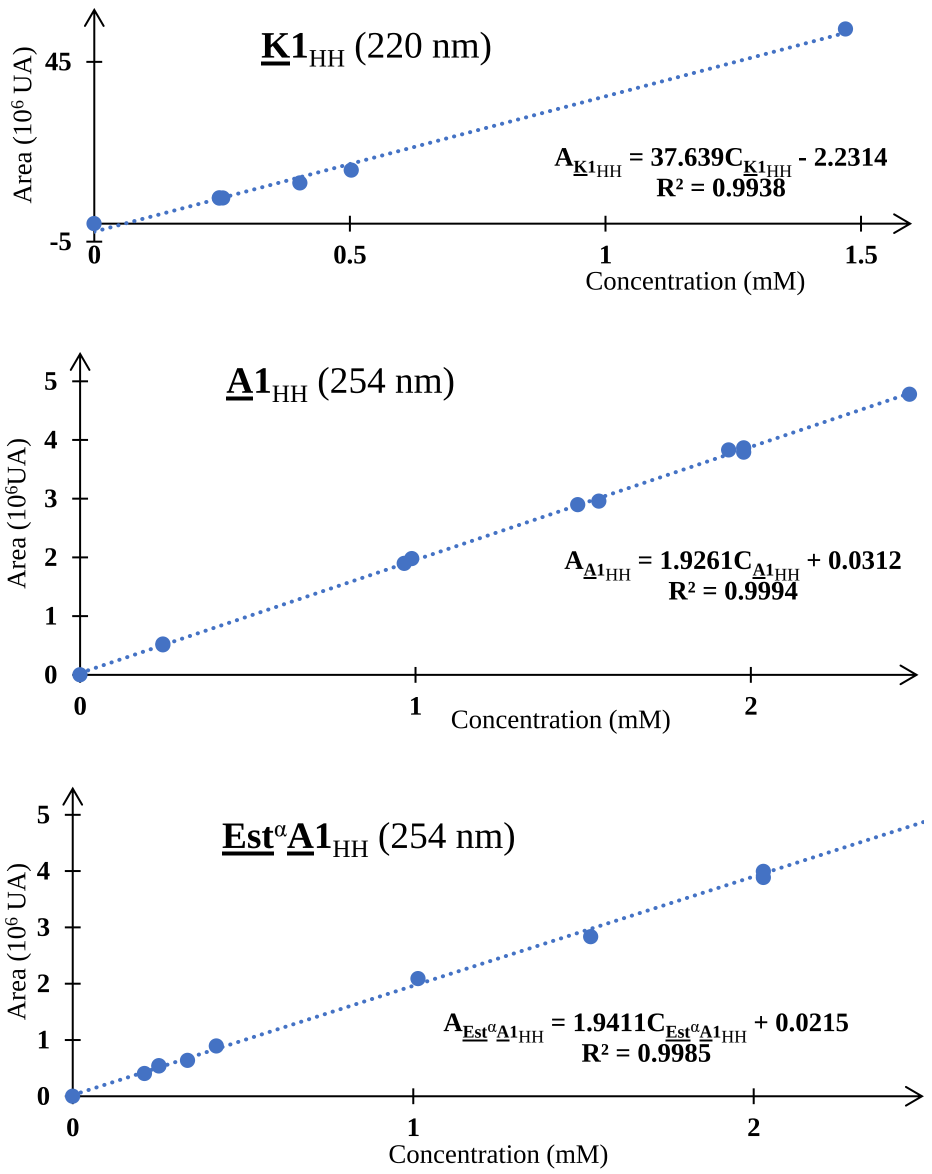

Figure S2. Calibration curves of the products and reactants in HPLC.

Table S1. List of all the products identified by HPLC.

Retention time (min)	Compound	Supplier	Comments	
4.7	*p*-quinone (99%)	Merck	Identification by a commercial standard<br>No calibration	Product from phenol oxidation
5.7	Mandelic acid (99%)	Aldrich		C-O cleavage product
8.0	Phenylglyoxylic acid (97%)	Aldrich		
8.3	Phenol (>99%)	Aldrich	Identification by a commercial standard.<br>Calibration at 210 nm	Main products from dimeric model oxidative cleavage
10.9	Benzaldehyde (>99%)	Aldrich		
13.0	2-phenoxyacetic acid	-	The standard was synthesized according to Ref S2<br>No calibration	Acetolysis product (non-oxidative C-O cleavage)
14.0	Benzoic acid (99%)	Aldrich	Identification by a commercial standard.<br>Calibration at 220 nm.	Main product from dimeric model oxidative cleavage
15.0	Acetophenone (>99%)	Fluka	Identification by a commercial standard<br>No calibration	Acidic C-O cleavage product
29.0	**D1**$_{HH}$<br>(E)-2-phenoxystyrene	**-**	Identification by NMR of a concentrated reaction media [S3].<br>Yield calculated at 254 nm.	**A1**$_{HH}$ dehydration product.
31.0	**K1**$_{HH}$<br>2-phenoxyacetophenone	-	Identification by synthesized standard according to Ref S1.<br>Calibration at 220 nm	Ketone substrate. **A1**$_{HH}$ oxidation product
34.0	**A1**$_{HH}$<br>2-phenoxy-1-phenylethanol	-	Identification by synthesized standard according to Refs S1 (**A1**$_{HH}$) and S2 (**Est**$^{\alpha}$**A1**$_{HH}$).<br>**Calibration at 254 nm**	Alcohol substrate
51.0	**Est**$^{\alpha}$**A1**$_{HH}$<br>Benzenemethanol, α-(phenoxymethyl)-, 1-acetate	-		Esterification product of **A1**$_{HH}$

- GC-MS

Table S2a. Gas chromatography–mass spectrometry (GC-MS) identification of products arising from C-O bond cleavage.

RT (min)	Compound	Area ratio (/PhOH) **K1**$_{HH}$ oxidation	**A1**$_{HH}$ oxidation	Match (%)	Comments
4.0	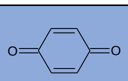 *p-quinone*	1 × 10^{-1}	1 × 10^{-2}	88	Identification by HPLC with a commercial standard. Minor product.
4.9	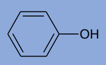 *phenol*	1	1	97	Targeted product. Identification by HPLC with a commercial standard.
6.3	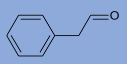 **phenylacetaldehyde**	-	1 × 10^{-2}	98	Acidic cleavage product.
6.8	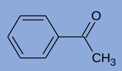 **acetophenone**	-	1 × 10^{-2}	91	Acidic cleavage product from phenylacetaldehyde isomerization.
11.2	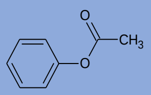 *Phenyl acetate*	-	5 × 10^{-2}	88	Phenol esterified by acetic acid
19.6	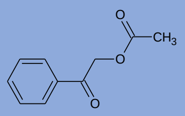 *2-acetoxyacetophenone*	5 × 10^{-1}	2 × 10^{-1}	95	Identification by HPLC with a synthesized standard. Acetolysis product.
phenol: product observed after **K1**$_{HH}$ cleavage, **phenylacetaldehyde : not observed after K1**$_{HH}$ **cleavage**					

Table S2b. GC-MS identification of products arising from C-C bond cleavage.

RT (min)	Compound	Area ratio (/PhCOOH) **K1**$_{HH}$ oxidation	**A1**$_{HH}$ oxidation	Match (%)	Comments
4.7	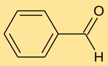 *Benzaldehyde*	4 × 10^{-3}	5 × 10^{-2}	96	Targeted product. **Identification by HPLC** with a commercial standard.
4.8	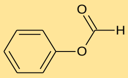 *Phenyl formate*	8 × 10^{-1}	4 × 10^{-2}	92	Formic ester of phenol.

9.8	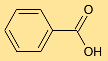 *Benzoic acid*	1	1	97	Targeted product. **Identification by HPLC** with a commercial standard.
17.2	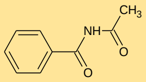 *N-acetylbenzamide*	9 × 10^{-2}	1 × 10^{-1}	93	Benzamide of benzoic acid and acetamide. Acetamide was also identified by GC-MS (3.93 min).
Benzaldehyde : product observed after **K1**HH cleavage					

Table S2c. Products with two aromatic units derived from **K1**HH and **A1**HH detected by GC-MS.

RT (min)	Compound	Area ratio (/**K1**HH) **K1**HH oxidation	**A1**HH oxidation	Match (%)	Comments
22.0	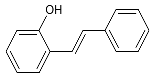 **2-hydroxystilbene**	-	3 × 10^{-3}	88	Oxidative condensation product from **A1**HH oxidation. Detected by ^{1}H NMR.
22.82	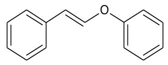 **D1**HH	-	2 × 10^{-3}	n. i.	Identified by GC-MS by the injection of a diluted reaction media (X 40) and ^{1}H NMR analysis of a concentrated reaction media [S4]. (Molecular peak at m/z = 196)
25.66	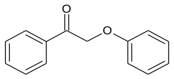 **K1**HH	1	1	n. i.	Identified by the injection of a diluted reaction media (Characteristic molecular peak at m/z = 212)
25.8	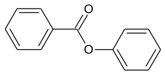 *Phenyl benzoate*	8 × 10^{-2}	3 × 10^{-2}	93	Ester of phenol and benzoic acid
27.0	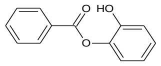 *2-hydroxyphenyl benzoate*	1 × 10^{-2}	4 × 10^{-2}	95–96	Oxidation side product
27.8	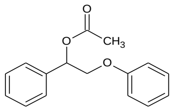 **Est**$^{\alpha}$**A1**HH	-	2 × 10^{-2}	n. i.	Peaks at m/z = 43 ($COCH_3^+$) and m/z = 196 (molecular peak of **D1**HH).
27.9	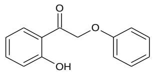 W	7 × 10^{-3}	-	n. i.	
28.7	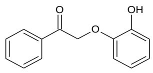 W′	5 × 10^{-3}	-	n. i.	

29.2	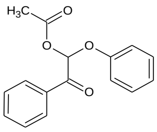 X	3×10^{-2}	n. i.	Structure supposed from GC-MS data of **<u>K1</u>**HH cleavage.

Phenyl benzoate: product observed after <u>K</u>1HH cleavage, 2-hydroxystilbene : not observed after **<u>K1</u>**HH cleavage

XRD profiles and Fullprof® refinement

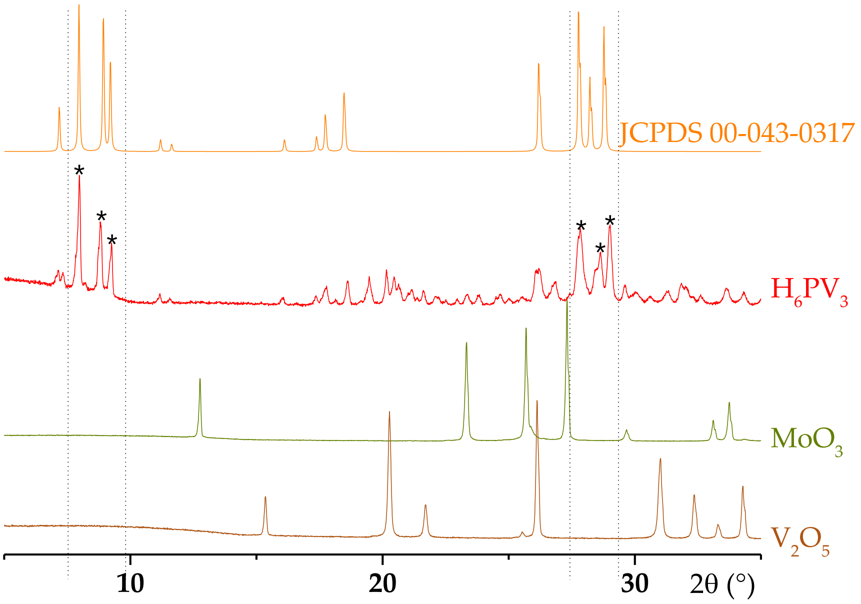

Figure S3. X-ray diffraction (XRD) profile of H_6PV_3 (main characteristic peaks marked by * symbol) vs. starting MoO_3 and V_2O_5 and reference H_3PMo_{12}.

A RIETVIELD analysis of the XRD profiles of H_6PV_3 was carried out on Fullprof®. The diffractogram of the catalyst was similar to the JCPDS file 00-043-0317. It corresponds to tridecahydrated phosphomolybdic acid. It has a triclinic crystalline structure (P-1). The main phase of H_6PV_3 has the same crystalline structure. On Figure S4 is given an extract of the H_6PV_3 Fullprof result:

```
*************************************************************
** PROGRAM FullProf.2k (Version 5.40 - Mar2014-ILL JRC) **
*************************************************************
M U L T I -- P A T T E R N
Rietveld, Profile Matching & Integrated Intensity
Refinement of X-ray and/or Neutron Data

Date: 18/01/2017    Time: 17:08:10.234

=> PCR file code: [redacted]
=> DAT file code: [redacted]_INSTR -> Relative contribution: 1.0000

==> CONDITIONS OF THIS RUN FOR PATTERN No.:   1
```

=> Global Refinement of X-ray powder diffraction data
=> Global Refinement of X-ray powder diffraction data
Bragg-Brentano(X-rays) or Debye-Scherrer geometry(Neutrons)
=> Title: [redacted]

=> Number of phases: 1
=> Number of excluded regions: 0
=> Number of scattering factors supplied: 0
=> Conventional weights: w=1.0/Variance(yobs)
=> Asymmetry correction as in J.Appl.Cryst. 26,128(1993)
=> Background linearly interpolated between the 10 points given
=> The 5th default profile function was selected
=> Pseudo-Voigt function (ETA variable)
X-parameter correspond to: ETA=ETA0+X*2theta
pV(x)= ETA*L(x)+(1-ETA)*G(x)

==> INPUT/OUTPUT OPTIONS:

=> Generate file *.PRF for plot
=> Output Integrated Intensities
=> Generate new input file *.PCR

=> Data supplied in free format for pattern: 1
=> Plot pattern at each cycle
=> Wavelengths: 1.54056 1.54439
=> Alpha2/Alpha1 ratio: 0.5000
=> Cos(Monochromator angle)= 1.0000
=> Asymmetry correction for angles lower than 0.000 degrees
=> Absorption correction (AC), muR-eff = 0.0000 0.0000
=> Base of peaks: 2.0*HW* 8.00

=> Number of cycles: 30
=> Relaxation factors ==> for coordinates: 1.00
=> for anisotropic temperature factors: 1.00
=> for halfwidth/strain/size parameters: 1.00
=> for lattice constants and propagation vectors: 1.00
=> EPS-value for convergence: 0.1
=> Background ==>

Position	Intensity	
5.10	1991.85	121.00
6.86	1720.11	131.00
7.20	1896.74	141.00

7.49	1625.00	151.00
8.14	1692.94	161.00
8.40	1475.54	171.00
9.00	1557.07	181.00
9.41	1163.04	191.00
9.87	945.65	201.00
10.86	809.78	211.00

=> Number of Least-Squares parameters varied: 21

=>-------------------------->
=>-------> PATTERN number: 1
=>--------------------------->
=> Global parameters and codes ==>
=> Zero-point: 0.0000 0.0000
=> Displacement peak-shift parameter and code: 0.00 0.00
=> Transparency peak-shift parameter and code: 0.00 0.00

=> Reading Intensity data =>>

==> Angular range, step and number of points:
2Thmin: 5.000000 2Thmax: 49.999737 Step: 0.009193 No. of points: 4896

=> Phase No. 1
[redacted]

=>-------> Pattern# 1
=> Profile Matching (fixed scale)
=> Input data if irf=2: Integrated Intensities
=> Preferred orientation vector: 0.0000 0.0000 1.0000

=>-------> Data for PHASE: 1
=> Number of atoms: 0
=> Number of distance constraints: 0
=> Number of angle constraints: 0

=> Symmetry information on space group: P -1
-> The multiplicity of the general position is: 2
-> The space group is Centric (-1 at origin)
-> Lattice type P: { 000 }
-> Reduced set of symmetry operators:

No. IT Symmetry symbol Rotation part Associated Translation
1: (1) 1 --> (x, y, z) + { 0.0000 0.0000 0.0000}

Information on Space Group:

=> Number of Space group: 2
=> Hermann-Mauguin Symbol: P -1
=> Hall Symbol: -P 1
=> Table Setting Choice:
=> Setting Type: IT (Generated from Hermann-Mauguin symbol)
=> Crystal System: Triclinic
=> Laue Class: -1
=> Point Group: -1
=> Bravais Lattice: P
=> Lattice Symbol: aP
=> Reduced Number of S.O.: 1
=> General multiplicity: 2
=> Centrosymmetry: Centric (-1 at origin)
=> Generators (exc. -1&L): 0

=> Asymmetric unit: 0.000 <= x <= 0.500

0.000 <= y <= 1.000

0.000 <= z <= 1.000

=> List of S.O. without inversion and lattice centring translations
=> SYMM(1): x,y,z

=>-------> PROFILE PARAMETERS FOR PATTERN: 1

=> Overall scale factor: 0.100000E-02
=> ETA (p-Voigt) OR M (Pearson VII): 2.7482
=> Overall temperature factor: 0.00000
=> Halfwidth U,V,W: 0.43634 -0.00878 -0.00031
=> X and Y parameters: -0.0731 0.0000
=> Direct cell parameters: 14.2195 14.4086 13.6151 112.5440 110.1295 60.1199
=> Preferred orientation parameters: 0.0000 0.0000
=> Asymmetry parameters : 0.00000 0.00000 0.00000 0.00000
=> Strain parameters : 0.00000 0.00000 0.00000

=> Size parameters : 0.00000 0.00000

==> CODEWORDS FOR PROFILE PARAMETERS of PATTERN# 1

=> Overall scale factor: 0.000
=> ETA (p-Voigt) OR M (Pearson VII): 81.000
=> Overall temperature factor: 0.000
=> Halfwidth U,V,W: 101.000 111.000 91.000
=> X and Y parameters: 71.000 0.000
=> Direct cell parameters: 11.000 21.000 31.000 41.000 51.000 61.000
=> Preferred orientation parameters: 0.000 0.000
=> Asymmetry parameters : 0.000 0.000 0.000 0.000
=> Strain parameters : 0.000 0.000 0.000
=> Size parameters : 0.000 0.000

=> Cell constraints according to Laue symmetry: -1

Metric information:

=> Direct cell parameters:

a =	14.2195	b =	14.4086	c =	13.6151
alpha =	112.544	beta =	110.129	gamma =	60.120

Direct Cell Volume = 2192.6455

=> Reciprocal cell parameters:

a*=	0.082633	b*=	0.082902	c*=	0.081020
alpha*=	74.910	beta*=	78.975	gamma*=	114.983

Reciprocal Cell Volume = 0.00045607

=> Direct and Reciprocal Metric Tensors:

	GD			GR	
202.1929	102.0698	-66.6259	0.006828	-0.002893	0.001280
102.0698	207.6088	-75.2121	-0.002893	0.006873	0.001749
-66.6259	-75.2121	185.3713	0.001280	0.001749	0.006564

=> Cartesian frame: x // a; y is in the ab-plane; z is x ^ y

Crystal_to_Orthonormal_Matrix Orthonormal_to_Crystal Matrix
Cr_Orth_cel Orth_Cr_cel

14.2195	7.1782	-4.6855		0.070326	-0.040407	0.015802
0.0000	12.4933	-3.3281		0.000000	0.080043	0.021583
0.0000	0.0000	12.3426		0.000000	0.000000	0.081020

Busing-Levy B-matrix: Hc=B.H				Inverse of the Busing-Levy B-matrix		
	BL_M				BL_Minv	
0.082633	-0.035013	0.015494		12.1017	5.6387	-4.8935
0.000000	0.075145	0.030489		0.0000	13.3076	-5.5242
0.000000	0.000000	0.073448		0.0000	0.0000	13.6151

=> Given Laue symmetry -1 Indices are read from file

=> The Number of Reflections read for phase 1 and pattern# 1 is: ***

SYMBOLIC NAMES AND INITIAL VALUES OF PARAMETERS TO BE VARIED:

-> Parameter number 1 -> Symbolic Name: Cell_A_ph1_pat1 14.219456
-> Parameter number 2 -> Symbolic Name: Cell_B_ph1_pat1 14.408635
-> Parameter number 3 -> Symbolic Name: Cell_C_ph1_pat1 13.615112
-> Parameter number 4 -> Symbolic Name: Cell_D_ph1_pat1 112.54399
-> Parameter number 5 -> Symbolic Name: Cell_E_ph1_pat1 110.12949
-> Parameter number 6 -> Symbolic Name: Cell_F_ph1_pat1 60.119942
-> Parameter number 7 -> Symbolic Name: X-tan_ph1_pat1 -0.73063999E-
01
-> Parameter number 8 -> Symbolic Name: EtaPV_ph1_pat1 2.7481799
-> Parameter number 9 -> Symbolic Name: W-Cagl_ph1_pat1 -
0.31300000E-03
-> Parameter number 10 -> Symbolic Name: U-Cagl_ph1_pat1 0.43633699
-> Parameter number 11 -> Symbolic Name: V-Cagl_ph1_pat1 -
0.87789996E-02
-> Parameter number 12 -> Symbolic Name: Bck_0_pat1 1991.8485
-> Parameter number 13 -> Symbolic Name: Bck_1_pat1 1720.1090
-> Parameter number 14 -> Symbolic Name: Bck_2_pat1 1896.7396
-> Parameter number 15 -> Symbolic Name: Bck_3_pat1 1625.0002
-> Parameter number 16 -> Symbolic Name: Bck_4_pat1 1692.9353
-> Parameter number 17 -> Symbolic Name: Bck_5_pat1 1475.5442
-> Parameter number 18 -> Symbolic Name: Bck_6_pat1 1557.0653
-> Parameter number 19 -> Symbolic Name: Bck_7_pat1 1163.0439
-> Parameter number 20 -> Symbolic Name: Bck_8_pat1 945.65222
-> Parameter number 21 -> Symbolic Name: Bck_9_pat1 809.78278

=> Zero counts at step no. 4896 at 2theta/TOF/E(KeV): 49.9997 Intensity fixed to 1.0 and variance to 1E6

=> No optimization for routine tasks
++

=> CYCLE No.: 18

=> Phase 1 Name: [redacted]

==> PROFILE PARAMETERS FOR PATTERN# 1

=> Overall scale factor: 0.001000000 0.000000000 0.000000000
=> Eta(p-Voigt) or m(Pearson VII): 2.669654 -0.005033 0.038337
=> Overall tem. factor: 0.000000 0.000000 0.000000
=> Halfwidth parameters:
0.132842 -0.011595 0.059931
0.103881 0.004534 0.021719
-0.005739 -0.000208 0.001335
=> Cell parameters:
14.218796 -0.000013 0.001226
14.409559 0.000032 0.001222
13.615664 0.000043 0.000928
112.542053 -0.000053 0.006520
110.133392 0.000702 0.007337
60.126492 0.000214 0.007890
=> Preferred orientation:
0.000000 0.000000 0.000000
0.000000 0.000000 0.000000
=> Asymmetry parameters:
0.000000 0.000000 0.000000
0.000000 0.000000 0.000000
0.000000 0.000000 0.000000
0.000000 0.000000 0.000000
=> X and Y parameters:
-0.070814 0.000005 0.001786
0.000000 0.000000 0.000000
=> Strain parameters:
0.000000 0.000000 0.000000
0.000000 0.000000 0.000000
0.000000 0.000000 0.000000
=> Size parameters (G,L):

0.000000 0.000000 0.000000
0.000000 0.000000 0.000000

==> GLOBAL PARAMETERS FOR PATTERN# 1

=> Zero-point: 0.0000 0.0000 0.0000
=> Background Parameters (linear interpolation) ==>

1969.03	0.553468E-03	20.0892
1713.08	-0.132889E-02	19.4847
2178.31	0.144182E-01	35.3255
1727.10	-0.580191E-01	28.0544
2527.98	0.547234	31.9704
1351.88	-2.79057	27.7499
835.487	-27.8055	28.9633
1050.55	-6.77594	24.3305
974.937	0.897318	15.7357
779.968	-1.40225	2.98687

=> Cos(theta)-shift parameter : 0.0000 0.0000 0.0000
=> Sin(2theta)-shift parameter : 0.0000 0.0000 0.0000

==> RELIABILITY FACTORS WITH ALL NON-EXCLUDED POINTS FOR PATTERN: 1

=> R-Factors: 5.50 9.92 Chi2: 12.0 DW-Stat.: 0.1993 Patt#: 1
=> Expected : 2.86 1.9199
=> Deviance : 0.797E+05 Dev*: 16.35
=> GoF-index: 3.5 Sqrt(Residual/N)
=> N-P+C: 4875

=>	SumYdif	SumYobs	SumYcal	SumwYobsSQ	Residual	Condition
	0.3282E+06	0.5963E+07	0.5918E+07	0.5963E+07	0.5863E+05	0.1054E+17

=> Conventional Rietveld Rp,Rwp,Re and Chi2: 19.7 27.7 7.98 12.03
=> (Values obtained using Ynet, but true sigma(y))
=> SumYnet, Sum(w Ynet**2): 0.1662E+07 0.7652E+06

==> RELIABILITY FACTORS FOR POINTS WITH BRAGG CONTRIBUTIONS FOR PATTERN: 1

=> R-Factors: 4.65 6.83 Chi2: 5.48 DW-Stat.: 0.4763 Patt#:

=> Expected : 2.92 1.9168
=> Deviance : 0.274E+05 Dev*: 6.119
=> GoF-index: 2.3 Sqrt(Residual/N)
=> N-P+C: 4480

=>	SumYdif	SumYobs	SumYcal	SumwYobsSQ	Residual	Condition
	0.2449E+06	0.5264E+07	0.5255E+07	0.5264E+07	0.2453E+05	0.1054E+17

=> Conventional Rietveld Rp,Rwp,Re and Chi2: 15.1 18.3 7.83 5.475
=> (Values obtained using Ynet, but true sigma(y))
=> SumYnet, Sum(w Ynet**2): 0.1626E+07 0.7311E+06

=> Global user-weigthed Chi2 (Bragg contrib.): 13.1

=> ---------> Pattern# 1
=> Phase: 1
=> Bragg R-factor: 1.35
=> RF-factor : 0.630

Standard deviations have to be multiplied by: 3.2459
(correlated residuals) See references:
-J.F.Berar & P.Lelann, J. Appl. Cryst. 24, 1-5 (1991)
-J.F.Berar, Acc. in Pow. Diff. II,NIST Sp.Pub. 846, 63(1992)

++

=> CYCLE No.: 18
=> Convergence reached at this CYCLE !!!!
=> Parameter shifts set to zero

=> Phase 1 Name: [redacted]

==> PROFILE PARAMETERS FOR PATTERN# 1

=> Overall scale factor: 0.001000000 0.000000000 0.000000000
=> Eta(p-Voigt) or m(Pearson VII): 2.669654 0.000000 0.038337
=> Overall tem. factor: 0.000000 0.000000 0.000000
=> Halfwidth parameters:
0.132842 0.000000 0.059931

```
      0.103881   0.000000   0.021719
     -0.005739   0.000000   0.001335
 => Cell parameters:
     14.218796    0.000000    0.001226
     14.409559    0.000000    0.001222
     13.615664    0.000000    0.000928
    112.542053    0.000000    0.006520
    110.133392    0.000000    0.007337
     60.126492    0.000000    0.007890
 => Preferred orientation:
      0.000000   0.000000   0.000000
      0.000000   0.000000   0.000000
 => Asymmetry parameters:
      0.000000   0.000000   0.000000
      0.000000   0.000000   0.000000
      0.000000   0.000000   0.000000
      0.000000   0.000000   0.000000
 => X and Y parameters:
     -0.070814   0.000000   0.001786
      0.000000   0.000000   0.000000
 => Strain parameters:
      0.000000   0.000000   0.000000
      0.000000   0.000000   0.000000
      0.000000   0.000000   0.000000
 => Size    parameters (G,L):
      0.000000   0.000000   0.000000
      0.000000   0.000000   0.000000

 ==> GLOBAL PARAMETERS FOR PATTERN#   1

 => Zero-point:     0.0000     0.0000     0.0000
 => Background Parameters (linear interpolation)   ==>
        1969.03          0.00000          20.0892
        1713.08          0.00000          19.4847
        2178.31          0.00000          35.3255
        1727.10          0.00000          28.0544
        2527.98          0.00000          31.9704
        1351.88          0.00000          27.7499
        835.487          0.00000          28.9633
        1050.55          0.00000          24.3305
        974.937          0.00000          15.7357
        779.968          0.00000          2.98687
```

=> Cos(theta)-shift parameter : 0.0000 0.0000 0.0000
=> Sin(2theta)-shift parameter : 0.0000 0.0000 0.0000

==> RELIABILITY FACTORS WITH ALL NON-EXCLUDED POINTS FOR PATTERN: 1

=> R-Factors: 5.50 9.92 Chi2: 12.0 DW-Stat.: 0.1993 Patt#: 1
=> Expected : 2.86 1.9199
=> Deviance : 0.797E+05 Dev*: 16.35
=> GoF-index: 3.5 Sqrt(Residual/N)
=> N-P+C: 4875

=> SumYdif	SumYobs	SumYcal	SumwYobsSQ	Residual	Condition
0.3282E+06	0.5963E+07	0.5918E+07	0.5963E+07	0.5863E+05	0.1054E+17

=> Conventional Rietveld Rp,Rwp,Re and Chi2: 19.7 27.7 7.98 12.03
=> (Values obtained using Ynet, but true sigma(y))
=> SumYnet, Sum(w Ynet**2): 0.1662E+07 0.7652E+06

=> N-sigma of the GoF: 544.410

==> RELIABILITY FACTORS FOR POINTS WITH BRAGG CONTRIBUTIONS FOR PATTERN:
1

=> R-Factors: 4.65 6.83 Chi2: 5.48 DW-Stat.: 0.4763 Patt#:
=> Expected : 2.92 1.9168
=> Deviance : 0.274E+05 Dev*: 6.119
=> GoF-index: 2.3 Sqrt(Residual/N)
=> N-P+C: 4480

=> SumYdif	SumYobs	SumYcal	SumwYobsSQ	Residual	Condition
0.2449E+06	0.5264E+07	0.5255E+07	0.5264E+07	0.2453E+05	0.1054E+17

=> Conventional Rietveld Rp,Rwp,Re and Chi2: 15.1 18.3 7.83 5.475
=> (Values obtained using Ynet, but true sigma(y))
=> SumYnet, Sum(w Ynet**2): 0.1626E+07 0.7311E+06

=> N-sigma of the GoF: 211.819

=> Global user-weigthed Chi2 (Bragg contrib.): 13.1

=> ---------> Pattern# 1
=> Phase: 1

=> Bragg R-factor: 1.36
=> RF-factor : 0.636

BRAGG R-Factors and weight fractions for Pattern # 1

=> Phase: 1 [redacted]
=> Bragg R-factor: 1.37 Vol: 2192.891(0.304) Fract(%): 0.00(0.00)
=> Rf-factor= 0.642 ATZ: 0.000 Brindley: 1.0000

SYMBOLIC NAMES AND FINAL VALUES AND SIGMA OF REFINED PARAMETERS:

-> Parameter number 1 : Cell_A_ph1_pat1 14.218796 (+/- 0.12258012E-02)
-> Parameter number 2 : Cell_B_ph1_pat1 14.409559 (+/- 0.12220141E-02)
-> Parameter number 3 : Cell_C_ph1_pat1 13.615664 (+/- 0.92841446E-03)
-> Parameter number 4 : Cell_D_ph1_pat1 112.54205 (+/- 0.65201637E-02)
-> Parameter number 5 : Cell_E_ph1_pat1 110.13339 (+/- 0.73373793E-02)
-> Parameter number 6 : Cell_F_ph1_pat1 60.126492 (+/- 0.78895539E-02)
-> Parameter number 7 : X-tan_ph1_pat1 -0.70813768E-01(+/- 0.17862907E-02)
-> Parameter number 8 : EtaPV_ph1_pat1 2.6696539 (+/- 0.38336717E-01)
-> Parameter number 9 : W-Cagl_ph1_pat1 -0.57393722E-02(+/- 0.13351092E-02)
-> Parameter number 10 : U-Cagl_ph1_pat1 0.13284162 (+/- 0.59930515E-01)
-> Parameter number 11 : V-Cagl_ph1_pat1 0.10388075 (+/- 0.21718623E-01)
-> Parameter number 12 : Bck_0_pat1 1969.0270 (+/- 20.089241)
-> Parameter number 13 : Bck_1_pat1 1713.0764 (+/- 19.484655)
-> Parameter number 14 : Bck_2_pat1 2178.3132 (+/- 35.325504)

-> Parameter number 15 : Bck_3_pat1 1727.0988 (+/- 28.054407
)
-> Parameter number 16 : Bck_4_pat1 2527.9788 (+/- 31.970407
)
-> Parameter number 17 : Bck_5_pat1 1351.8751 (+/- 27.749874
)
-> Parameter number 18 : Bck_6_pat1 835.48700 (+/- 28.963268
)
-> Parameter number 19 : Bck_7_pat1 1050.5460 (+/- 24.330479
)
-> Parameter number 20 : Bck_8_pat1 974.93671 (+/- 15.735664
)
-> Parameter number 21 : Bck_9_pat1 779.96844 (+/- 2.9868696
)

=> Number of bytes for floating point variables: 4

=> Dimensions of dynamic allocated arrays in this run of FullProf:

=> Total approximate array memory (dynamic + static): 92103993 bytes

MaxPOINT= 60000 Max.num. of points(+int. Inten.)/diffraction pattern

MaxREFLT= 20000 Max.num. of reflections/diffraction pattern

MaxPARAM= 300 Max.num. of refinable parameters

MaxOVERL= 2096 Max.num. of overlapping reflections

=> Number of bytes for floating point arrays: 4

=> Dimensions of fixed arrays in this release of FullProf:

NPATT = 80 Max.num. of powder diffraction patterns

NATS = 830 Max.num. of atoms (all kind) in asymmetric unit

MPAR = 800 Max.num. of non atomic parameters/phase

IEXCL = 30 Max.num. of excluded regions

IBACP = 277 Max.num. of background points for interpolation

NPHT = 16 Max.num. of phases

NMAGM = 8 Max.num. of rotation-matrices sets for magnetic structure

NBASIS = 12 Max.num. of basis functions associated to a single atom

NIREPS = 9 Max.num. of irreducible representations to be combined

N_EQ = 384 Max.num. of user-supplied symmetry operators/propagation vectors

NGL = 300 Max.num. of global parameters/diffraction pattern

N_LINC = 30 Max.num. of global linear restraints
NAT_P = 64 Max.num. of atomic parameters per atom
NCONST = 500 Max.num. of slack constraints per phase
N_SPE = 16 Max.num. of different chemical species
N_FORM = 60 Max.num. of scattering factor values in a table
NPR = 150 Max.num. of points defining a numerical profile
INPR = 25 Max.num. of different numerical peak shapes
NPRC = 150 Max.num. of terms in the table for correcting intensities
NSOL = 10 Max.num. of solutions to be stored in Montecarlo searchs

Figure S4. Extract of Fullprof result of H_6PV_3 catalyst.

-

Choice of the ^{31}P NMR relaxation delay for P quantification in H_6PV_3

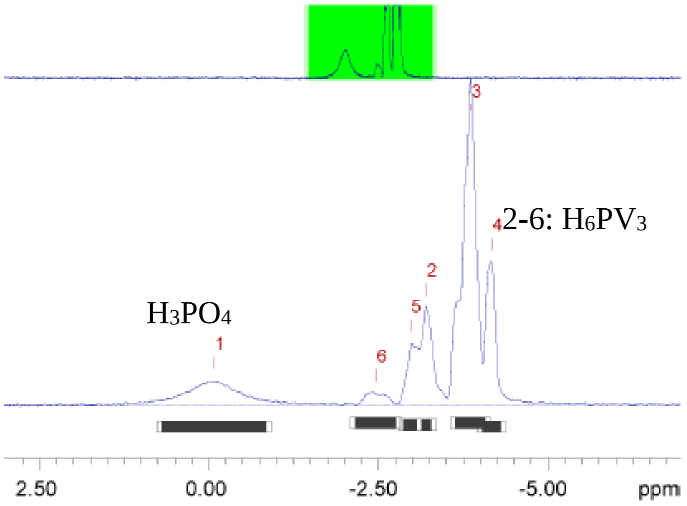

Figure S5. Spectrum of H_6PV_3.

NB: The pH is more acidic as 300 mg of H_6PV_3 was used instead of 30 mg. Since the position of the peaks depends on the pH, the phosphorous center in H_6PV_3 [S5] in those conditions was more deshielded (vs Figure 4).

The peak at 0 ppm corresponds to H_3PO_4. The peak n 6 corresponds to $H[PMo_9V_3O_{40}]^{5-}$ and the peaks 2–5 correspond to $[PMo_9V_3O_{40}]^{6-}$ and other $H_{3+x}PV_x$ (x < 3) [S5]. The order of the peaks is unchanged compared to typical ^{31}P NMR analysis (30 mg $H_{3+x}PV_x$). The longest relaxation delay is 1.02 s. Thus, the minimal relaxation time should be 6.1 s. So, as the relaxation time in typical ^{31}P NMR analysis was 32 s, the integration is quantitative.

[redacted]

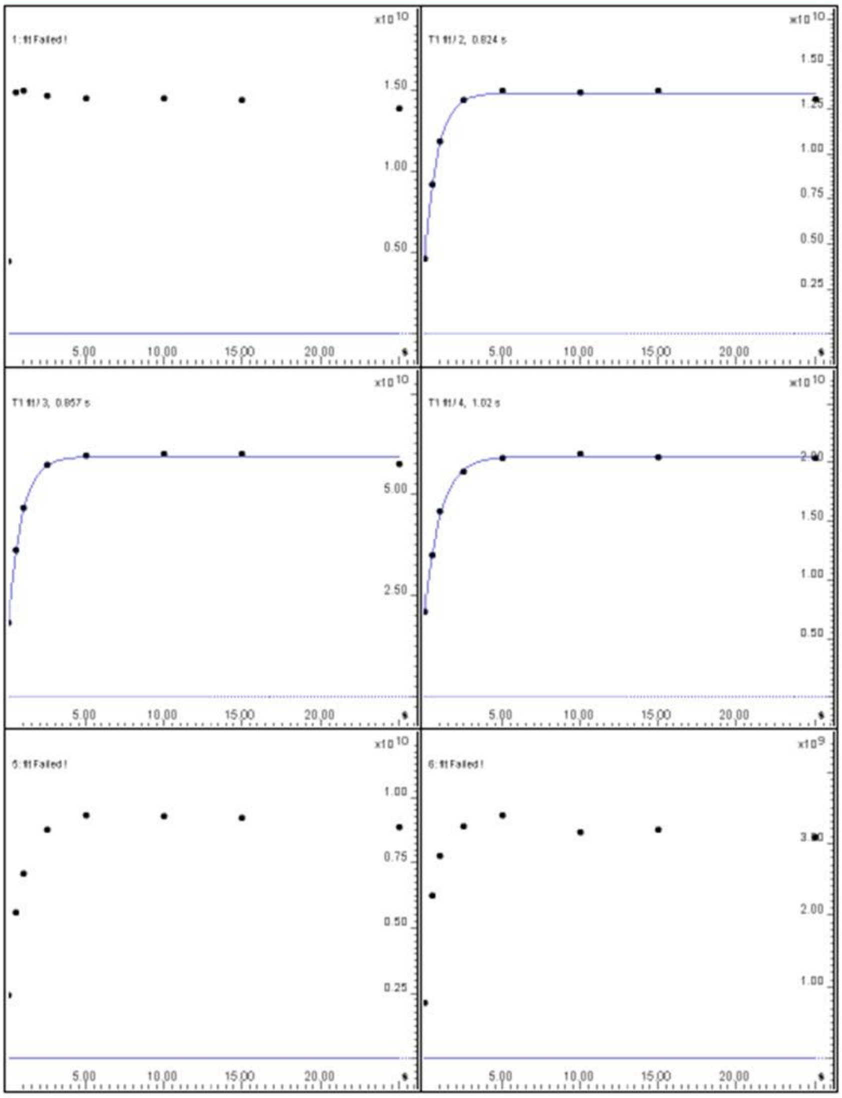

Fitted function:	f(t) = Io * [1 - a*exp (-t/T1)]
Random error estimation of data:	RMS per spectrum (or trace/plane)
Systematic error estimation of data:	worst case per peak scenario
Fit parameter Error estimation method:	from fit using arbitray y uncertainties
Confidence level:	95%
Used peaks:	
Used integrals:	area integral
Used Mixing time:	all values (including replicates) used

Peak name	F2 [ppm]	T1 [s]	error
1	-0.080	0.00	0.000
2	-3.209	0.824	0.1096
3	-3.864	0.857	0.1271
4	-4.171	1.02	0.1137
5	-2.969	0.00	0.000
6	-2.464	0.00	0.000

Figure S6. Graphical relaxation time calculation.

Kinetics

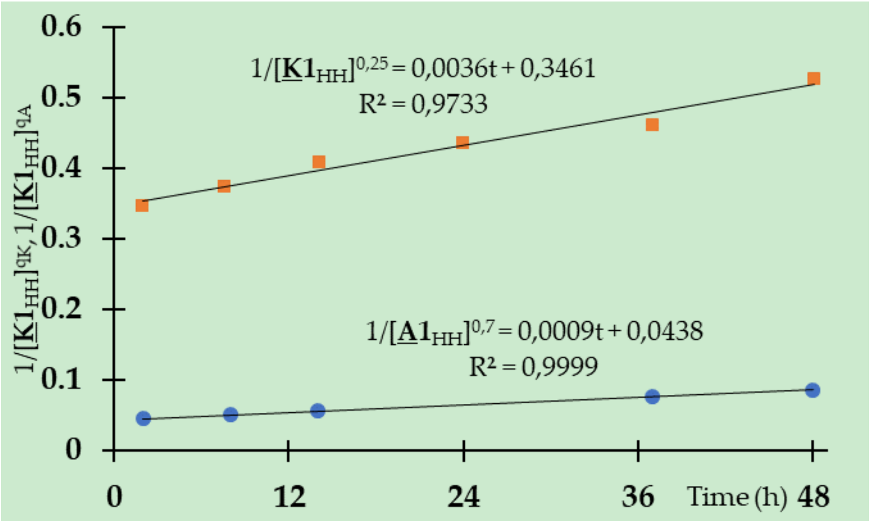

Figure S7. Graphical calculation of the kinetic order to K1HH (qK = 1.25, T = 80 °C) and A1HH (qA = 1.7, T = 80 °C).

Calibration curves of phenolic aldehydes and acids from lignin oxidative depolymerization

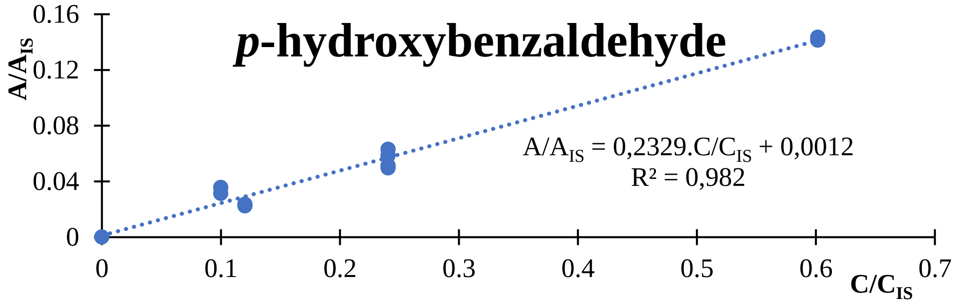

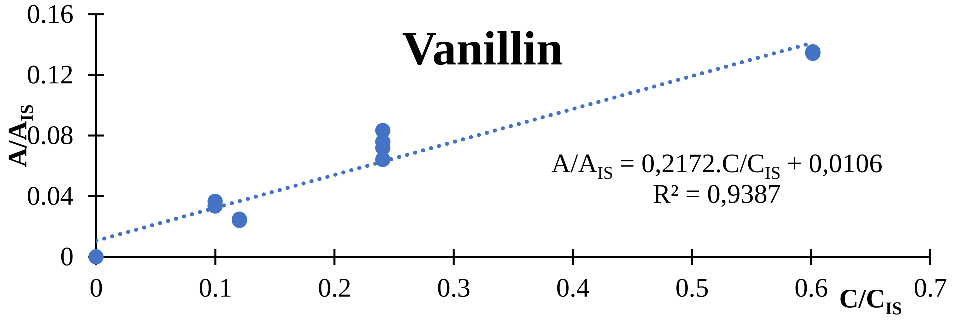

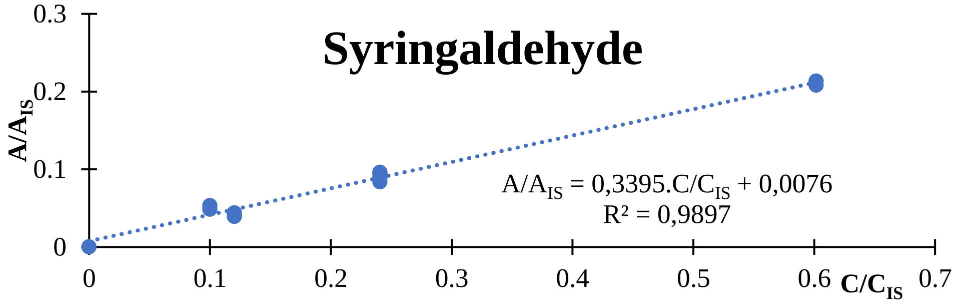

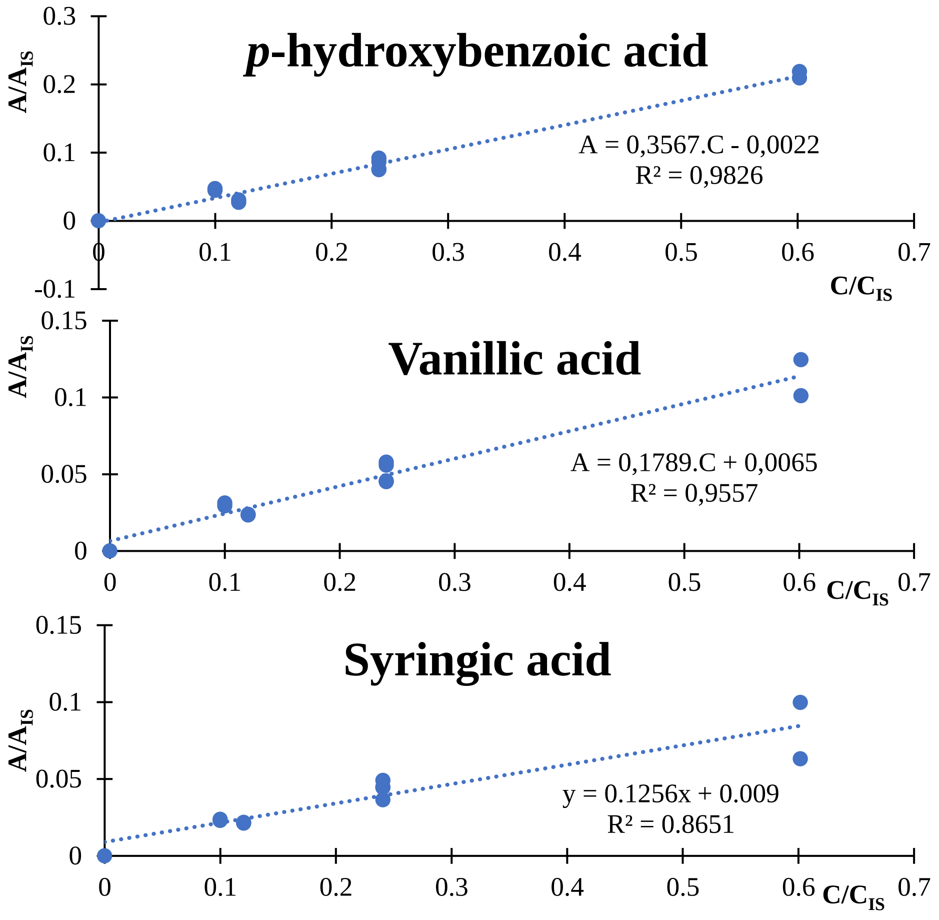

Figure S8. Calibration curves of the targeted silylated phenolic aldehydes and acids.

Impact of acetic acid on dioxygen solubility

In the case of a mixture of acetonitrile (MeCN) and acetic acid (AcOH), intuitively, a raise of the volumic fraction of acetic acid noted v should enhance the dioxygen solubility since dioxygen is more soluble in AcOH. The starting equation (Equation S1) [S6] is:

$$R^2_{mix}(v) = 4(\delta_{d,mix}(v) - \delta_{d,O_2})^2 + (\delta_{p,mix}(v) - \delta_{p,O_2})^2 + (\delta_{h,mix}(v) - \delta_{h,O_2})^2 = \sum_i a_i R_{j,i}(v)^2. \quad (1)$$

The δ_d, δ_p and δ_h terms stand for the dispersion forces, the dipole interaction and hydrogen bonding between the solvent j and dioxygen dissolved, $R_{j,i} = (\delta_{j,i} - \delta_{j,O_2})$ denotes the difference of solubility parameters (solvent *vs* dioxygen) and a_i is the multiplying factor of $R_{j,i}^2$.

For a mixture of two solvents s_1 (volumic fraction v_1) and s_2 (volumic fraction v_2):

$$\forall i, \forall(s_1, s_2), \delta_{i,mix\,(s_1,s_2)} = v_1\delta_{i,s_1} + v_2\delta_{i,s_2} = v_1(\delta_{i,s_1} - \delta_{i,s_2}) + \delta_{i,s_2}. \quad (2)$$

Therefore, the equation (Equation S3) is obtained from the application of (Equation S2) to the binary acetonitrile (MeCN) – acetic acid (AcOH):

$$\forall v \in [0;1]\ \delta_{i,mix\,(MeCN,AcOH)} = v\delta_{i,AcOH} + (1-v)\delta_{i,MeCN} = v_1(\delta_{i,s_1} - \delta_{i,s_2}) + \delta_{i,s_2}. \quad (3)$$

As acetonitrile and acetic acid are miscible at each proportion, it can be supposed that:

$$\forall v \in [0;1] \sum_i a_i(\delta_{i,AcOH} - \delta_{i,MeCN})^2 \ll \sum_i a_i(v\delta_{i,AcOH} - (1-v)\delta_{i,MeCN} - \delta_{i,O_2})^2 \quad (4)$$

$$\forall v \in [0;1]\ R^2_{mix}(v) \approx vR^2_{AcOH} + (1-v)R^2_{MeCN}\ . \quad (5)$$

So, The solubility can be estimated using this equation from Reference S4:

$\forall v \in [0; 1]\ \log(x_G) = -0.0889 R_{mix}(v) - 1.10$ (Equation S6), where x_G is the molar fraction of dioxygen at the maximum of solubility.

The predicted evolution of O_2 solubility determined from (Equation S5) and (Equation S6) in function of the volumic fraction of acetic acid is plotted on Figure S6.

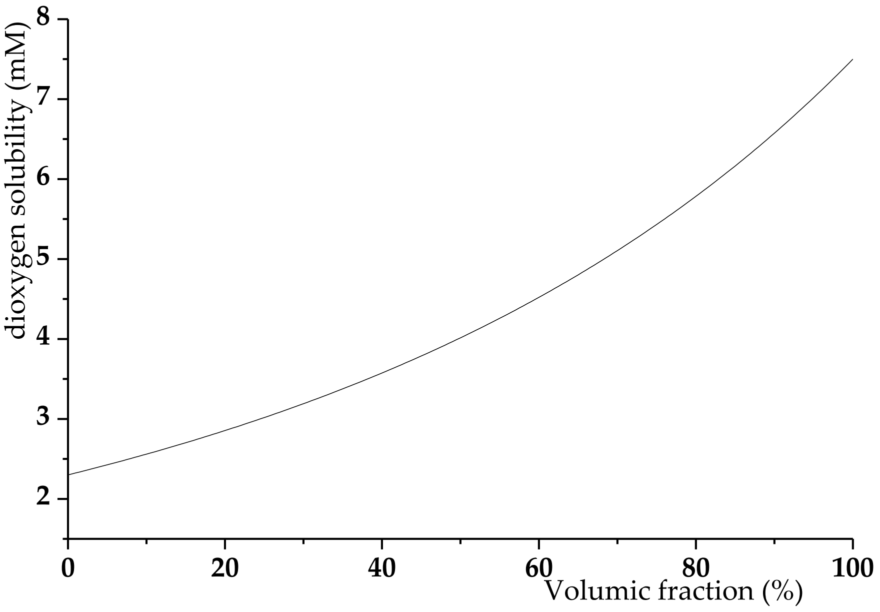

Figure S9. Evolution of O_2 solubility in function of the acetic acid content.

Lignin aerobic cleavage

Table S3. List of H_6PV_3 catalyzed lignin aerobic oxidative cleavage detected by GC-MS.

RT (min)	Compound (Match%)	GC-MS area (/vanillin)	Structure	Comments
4.0	*p*-quinone (83%)	0.03	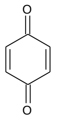	From C(Ar)-C cleavage
11.3	methoxy-*p*-quinone	0.36	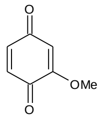	Supposed from MS data
14.2	*p*-hydroxybenzaldeyde (H) (97%)	0.28	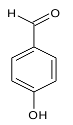	From C^{α}-C^{β} cleavage. Identified by the injection of a commercial sample.
14.6	Silylated H	-	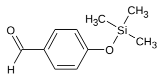	Calibrated by GC-MS. Identified by the injection of a silylated commercial sample
15.3	Vanillin (V) (97%)	1	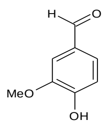	From C^{α}-C^{β} cleavage. Identified by the injection of a commercial sample.

18.8	Silylated V	-	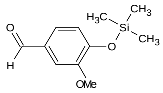	Calibrated by GC-MS. Identified by the injection of a silylated commercial sample
19.5	Vanillic acid (VA) (95%)	0.05	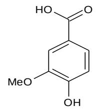	From C^{α}-C^{β} cleavage. Identified by the injection of a commercial sample.
19.7	2,6-dimethoxybenzoquinone (2,6-DMBQ) (92%)	1.3	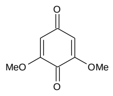	From C(Ar)-C cleavage.
20.1	Acetovanillone (84%)	0.3	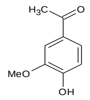	From C^{β}-O cleavage.
21.0	Silylated HA (*p*-hydroxybenzoic acid) (97%)	-	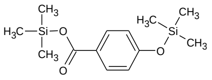	Calibrated by GC-MS. Identified by the injection of a silylated commercial sample
22.8	Silylated S (syringaldehyde) (97%)	-	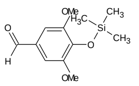	
24.2	Silylated VA	-	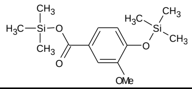	
27.1	Silylated SA (syringic acid)	-	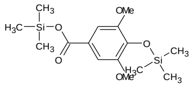	

Lignin 0.854 g, H_6PV_3 (Mo + V 15 mol%), O_2 5 bar, 120°C, 6 h (Table 4, entry 3)

Mechanistic studies

As mentioned in Table S2c, the structure proposed by the NIST Library (X′) was not satisfying. Further investigations had to be done by comparing in particular the masses of the different fragments of X with those of **K1**$_{HH}$ (Table 4).

Table S4. Mass table of the compound X and **K1**$_{HH}$.

Compound X	105 (100); 94 (43.9); 43 (42.8); 77 (33.8); 51 (9.7); 227 (8.6); 106 (7.9); 136 (5.3); 95 (4.5); 66 (3.2)
**K1**$_{HH}$	105 (100); 77 (63.3); 212 (43.0); 106 (17.3); 51 (15.6); 65 (7.7); 91 (7.2); 213 (6.8); 78 (4.7); 39 (4.7)
**X′**	105 (100); 77 (37.3); 51 (10.0); 106 (6.1); 43 (4.6); 50 (2.9); 78 (2.5); *240 (2.1)*; 39 (209); 76 (2.0)
**W**	*121 (100)*; 39 (23.9); 65 (22.8); 77 (21.6); 51 (12.0); 228 (10.5); 93 (10.3); *122 (7.3)*; 63 (6.0); 53 (4.8)
**W′**	105 (100); 77 (34.3); 106 (7.9); 51 (5.1); 78 (2.1); 50 (1.3); 76 (1.3); *214 (1.1)*; 52 (0.6); 107 (0.6)

Indeed, the molecular peak (m/z = 240) of the reference compound X′ proposed by the NIST Library was absent in the mass spectrum of the compound X whereas the peaks at m/z = 94 ($PhOH^{\cdot+}$)

and m/z = 136 ($PhCOCHO^{\cdot+}$, may be obtained from the cleavage of O-Ac and C-OPh bonds) were only observed for X and not X′. Besides, the peak at m/z = 227 may correspond to an oxyradical from hydroxylated **K1**$_{HH}$. The peak at m/z = 121 ($HOPhCHO^{+}$) was not observed for X unlike the compound W whereas the peak at 94 was observed unlike the compound W′. Moreover, the peak at m/z = 136 ($PhCH_2OH^{+}$) is observed only for X only. As an intense peak is observed at m/z = 43 is present, it is probable the hydroxyl group is acetylated.

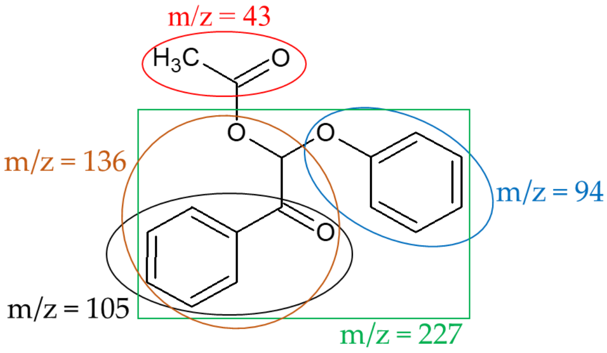

Figure S10. Hypothetic structure of the intermediate X.

Lignin phosphorylation

The phosphorylation was carried out according to the method of GRANATA and ARGYROPOULOS with slight modifications [S7,S8] using pyridine-$CDCl_3$ 1.6-1 as the solvent.

30 mg of lignin were dissolved in 500 µL of pyridine-$CDCl_3$ 1.6-1. Then, 100 µL of chromium acetylacetonate (III) 0.014 M and 100 µL of N-hydroxy-6-norbornene-2,3-dicarboximide 0.1 M (internal standard, 151.9 ppm) both in pyridine-$CDCl_3$ 1.6-1 were added followed by 150 µL of 2-chloro-4,4,5,5-tetramethyl-1,3,2-dioxaphospholane to start the phosphorylation reaction. The mixture was stirred overnight. The involved reaction is described in Figure S6.

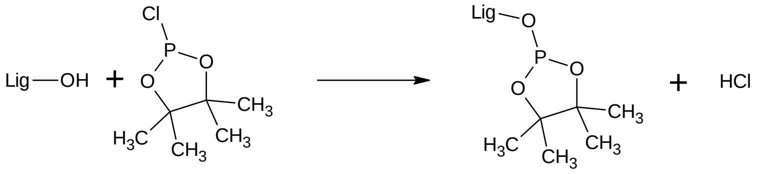

Figure S11. Equation of lignin phosphorylation.

Before the reaction starts, the purified lignin sample is not totally soluble in the solvent but usually the reaction leads to the dissolution of the solid. An example of the obtained ^{31}P NMR spectra is given on Fig. S7. This was not observed for oxidized lignin samples. So, the total content of OH functions could not be determined in these conditions.

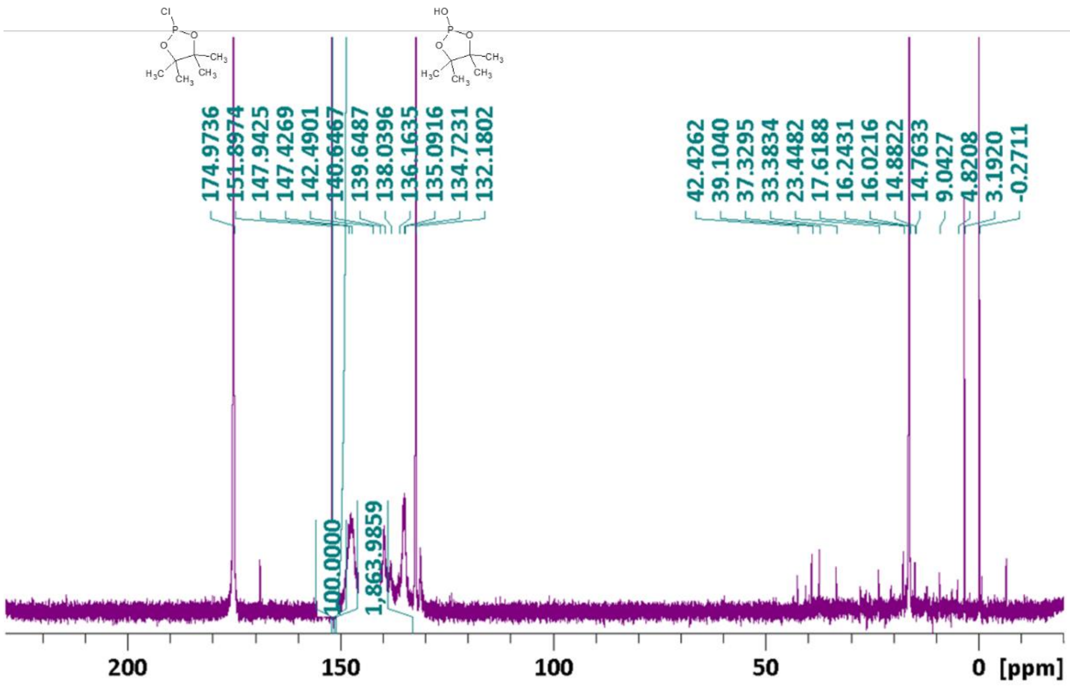

Figure S12a. ^{31}P NMR spectrum of phosphorylated non-oxidized lignin.

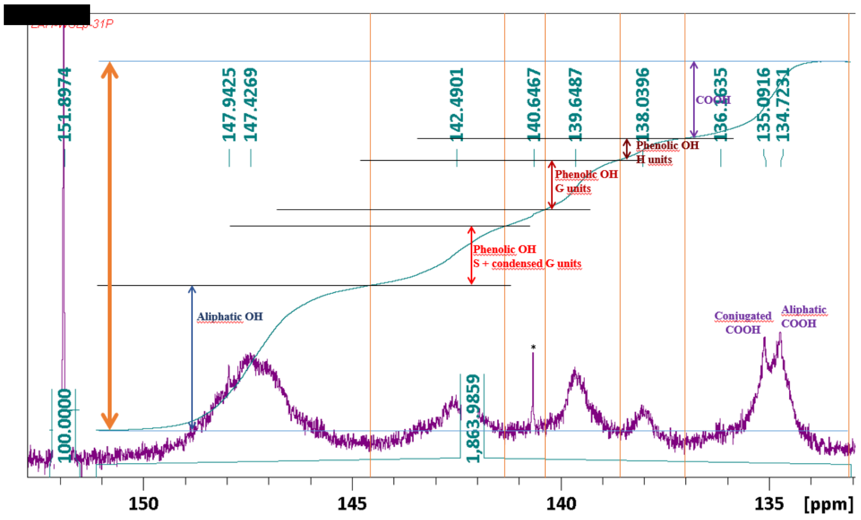

Figure S12b. Division of the interesting region of the 31P NMR spectrum of phosphorylated lignin for the quantification of the different types of OH groups (The internal standard was N-hydroxy-6-norbornene-2,3-dicarboximide (151.9 ppm)).

Table S5. Attribution of OH functions.

Chemical shift (ppm)	OH type
144.5–151	Aliphatic
141–144.5	Phenolic (S + condensed G units)
138.5–140.5	Phenolic (uncondensed G units)
137–138.5	Phenolic (H units)
133–137	Carboxylic acid (unconjugated 134.5 ppm and conjugated 134 ppm)

References

S1. Nichols, J. M.; Bishop, L. M.; Bergman, R. G.; Ellman J. A. Catalytic C-O Bond Cleavage of 2-Aryloxy-1-arylethanols and Its Application to the Depolymerization of Lignin-Related Polymers, *J. Am. Chem. Soc.* **2010**, *132*, 12554–12555, doi: 10.1021/ja106101f.

S2. Delmas, G-H.; Benjelloun-Mlayah, B.; Le Bigot, Y.; Delmas, M. Functionality of wheat straw lignin extracted in organic acid media, *J. Appl. Polym. Sci.* **2011**, *121*, 491–501. doi: 10.1002/app.33592.

S3. Zhang, C.; Lu, J.; Zhang, X.; MacArthur, K.; Heggen, M.; Li, H.; Wang, F.; Zhang, C.; Lu, J.; Zhang, X.; MacArthur, K.; Heggen, M.; Li, H.; Wang, F. Cleavage of the lignin β-O-4 ether bond via a dehydroxylation–hydrogenation strategy over a NiMo sulfide catalyst. *Green Chem.* **2016**, *18*, 6545–6555, doi: 10.1039/C6GC01456A

S4. Lion, C.J.; Matthews, C.S.; Stevens, M.F.G.; Westwell, A.D. Identification of a terphenyl derivative that blocks the cell cycle in the G0-G1 phase and induces differentiation in leukemia cells. *J. Med. Chem.* **2005**, *48*, 1292–1295.

S5. Pettersson L. Equilibria of Polyoxometalates in Aqueous Solution, *Mol. Eng.* **1993**, *3*, 29–42, doi:10.1007/BF00999622.

S6. Sato, T.; Hamada, Y.; Sumikawa, M.; Araki, S.; Yamamoto, H. Solubility of oxygen in organic solvents and calculation of the Hansen solubility parameters of oxygen. *Ind. Eng. Chem. Res*. **2014**, *53*, 19331–19337, doi: 10.1021/ie502386t.

S7. Granata, A.; Argyropoulos, D.S. 2-Chloro-4,4,5,5-tetramethyl-1,3,2-dioxaphospholane, a Reagent for the Accurate Determination of the Uncondensed and Condensed Phenolic Moieties in Lignins. *J. Agric. Food. Chem.* **1995**, 43, 1538–1544, doi: 10.1021/jf00054a023.

S8. Mbotchak, L.; Le Morvan, C.; Linh Duong, K.; Rousseau, B.; Tessier, M.; Fradet, A. Purification, Structural Characterization, and Modification of Organosolv Wheat Straw Lignin, *J. Agric. Food Chem.* **2015**, 63, 5178–5188, doi: 10.1021/acs.jafc.5b02071.

© 2020 by the authors. Submitted for possible open access publication under the terms and conditions of the Creative Commons Attribution (CC BY) license (http://creativecommons.org/licenses/by/4.0/).
